# The CsLOB1‐CsERF027 Regulatory Module Positively Enhances Citrus Target Spot Disease Resistance by Regulating CsRAP2.3‐CsERF1 Cascade

**DOI:** 10.1111/pbi.70306

**Published:** 2025-08-28

**Authors:** Fengjiao Liu, Siyu Long, Junhua Hu, Xinghua Qiao, Li Chen, Yan Zhou, Xiuping Zou

**Affiliations:** ^1^ Citrus Research Institute Southwest University Chongqing China; ^2^ Plant Protection and Fruit Tree Technology Popularization Station in Wanzhou District of Chongqing City Chongqing China

**Keywords:** CsLOB1, disease resistance, JA/ET signal pathway, *Pseudofabraea citricarpa*

## Abstract

Citrus target spot disease poses significant threats to the citrus industry. However, the molecular mechanisms underlying citrus resistance to *Pseudofabraea citricarpa* remain unexplored. In this study, we identified the LBD family gene *CsLOB1* and investigated its molecular responses to pathogen infection. Following *P. citricarpa* inoculation, exogenous methyl jasmonate (MeJA) and ethylene (ET), *CsLOB1* expression levels in ‘Eureka’ citrus were significantly higher than in ‘Tarocco’ blood orange. OE‐*CsLOB1* transgenic *Citrus sinensis* exhibited reduced susceptibility to *P. citricarpa* concomitant with enhanced expression of systemic acquired resistance marker genes (*CsPR1*, *CsPR2* and *CsPR4*), maintaining reactive oxygen species (ROS) homeostasis and reductions in H_2_O_2_ and malondialdehyde (MDA) accumulation. Conversely, *CsLOB1*‐RNAi lines exhibited exacerbated oxidative damage that aggravated the severity of citrus disease. Yeast one‐hybrid, yeast two‐hybrid and bimolecular fluorescence complementation (BiFC) assays confirmed that CsLOB1 directly binds to the CsRAP2.3 promoter and the heterodimer formation between *CsLOB1* and *CsERF027* to upregulate its expression. CsRAP2.3 physically interacts with the CsERF1 promoter, activating the key marker gene *CsPDF1.2* in the JA/ET‐dependent ERF/ORA59 signal pathway. Notably, exogenous MeJA application increased citrus resistance, and *P. citricarpa* infection significantly increased the content of JA in citrus. Virus‐induced gene silencing (VIGS)‐mediated silencing of *CsRAP2.3*, *CsERF1* or *CsERF027* enhances the citrus disease incidence. Conversely, overexpression of *CsRAP2.3*, *CsERF1* or *CsERF027* in transgenic lines conferred higher disease resistance. Mechanistically, this study establishes that CsLOB1 forms a transcriptional complex with CsERF027 through protein–protein interactions, synergistically activating the CsRAP2.3‐CsERF1 regulatory cascade. This coordinated regulation ultimately triggers the JA/ET‐dependent ERF/ORA59 branch pathway.

## 1 Introduction

The citrus target spot disease (TSD) is a low‐temperature‐induced disease caused by the fungus *Pseudofabraea citricarpa*; it will form a rotostriate lesion after infecting citrus tissues (Chen et al. 2016; Zhu et al. 2012). The *P. citricarpa* can harm the branches, leaves, treetops, stems, flowers and fruits of citrus, seriously threatening the production of citrus (Zhan et al. 2021; Zhan et al. 2024). Citrus TSD was first discovered and reported in the Chenggu citrus‐producing area of Shaanxi Province in 2012 (Zhu et al. 2012). This disease continued to spread for several years, causing more than 18 600 hm^2^ of affected area in Shaanxi citrus‐producing areas (Zhan et al. 2024; Zhu et al. 2012). In 2018, citrus TSD spread to Wanzhou, Chongqing, infecting many citrus × limon ‘Eureka’ (Eureka) varieties (Zhan et al. 2021). In 2020, the occurrence of TSD was reported in the citrus‐producing areas of Hubei and Hunan, indicating that citrus TSD is spreading from north to south (Xiao et al. 2020). Studies have shown that TSD is a low‐temperature pathogenic fungal disease, contrary to the characteristics of common citrus fungal diseases (such as *Citrus anthracnose* and *Alternaria* brown spot) in the high‐temperature season (Liu et al. 2024; Timmer et al. 2000; Wang, Chi, et al. 2021). With the aggravation of the disease, the leaves will wither, branches will wither and dry, and the incidence rate of citrus TSD in orchards was 100%, with the disease index reaching 60%–70% (Chen et al. 2023). At present, chemical agents are the main methods to prevent and control TSD, but long‐term use of chemical pesticides will not only cause pathogen resistance but also cause a lot of environmental pressure (Liu, Qiao, et al. 2023). Therefore, the breeding of disease‐resistant citrus varieties is the most economical, green and efficient research direction and strategy for the prevention and control of TSD (Chen et al. 2022). However, how citrus regulates its endogenous environment to resist the growth and reproduction of pathogens in the host during the interaction with *P. citricarpa*, as well as the molecular mechanism affecting the infection degree of *P. citricarpa* in different citrus varieties, remains unknown.

Studies have shown that plants are coupled by hormone signalling to form a complex regulatory network, which plays a crucial role in plant disease resistance. Among them, salicylic acid, jasmonic acid, ethylene and other plant hormones are widely involved in the resistance of plants to pathogens. Jasmonic acid (JA) and its related metabolites are lipid‐derived compounds, which are important substances for plants to cope with pathogen infection (Gfeller et al. 2010; Yang et al. 2023). The JA‐dependent signalling pathway in potato is activated by the infection of *P. infestans*, which induces hypersensitivity (HR) response through the accumulation of ROS and activates the expression of systematically acquired resistance marker genes such as PR to resist pathogen invasion (Halim et al. 2009). In *Arabidopsis thaliana* , two branches related to the JA signalling pathway have been identified: one is the JA/ET signalling pathway that relies on ERF1/ORA59 transcription factors and the JA response marker gene *PDF1.2*, and the other is controlled by *MYC* transcription factors, including the JA response marker gene *VSP2* (Berrocal‐Lobo et al. 2002; Dombrecht et al. 2007; McGrath et al. 2005). The ethylene (ET), salicylic acid (SA) and jasmonic acid (JA) signalling pathways in plant disease resistance networks share numerous overlapping components and interactions (Sato et al. 2010). In plants, SA and JA pathways have antagonistic effects, and ET is an important signalling molecule regulating the balance between these two signalling pathways, which can enhance the resistance response mediated by *SA*/*NPR1* and reduce the inhibition of SA on the resistance genes *PDF1.2* and *VSP2* induced by MeJA (Leon‐Reyes et al. 2009).

The *AP2*/*ERF* (APETALA2/ethylene responsive factor) transcription factor family contains 1–2 *AP2*/*ERF* domains composed of about 60 amino acids. It contains five subfamilies: *AP2* (APETALA2), ethylene response factor (*ERF*), dehydration response element‐binding protein (DREB), RAV and Soloist (Nakano et al. 2006). *AP2/ERF* transcription factors typically interact with various cis‐elements, including the GCC‐box, dehydration response element (DRE) and C‐repeat binding factor (CBF), found in the promoters of target genes. These interactions play a crucial role in plant responses to biotic stress by activating or inhibiting the expression of downstream genes (Zafar et al. 2022). Studies have shown that *CsAP2‐09‐CsWRKY25* enhances resistance to citrus canker by transcriptionally activating ROS accumulation mediated by *CsRBOH2* (Li et al. 2024). *MdERF114* can promote lignin deposition in apple tree roots by binding to the MdPRX63 promoter and regulating its transcription, thereby improving plant resistance to *Fusarium solani* (Liu, Liu, et al. 2023). *ERF* members of the *AP2*/*ERF* transcription factor family have been shown to act as key regulatory centres integrating ethylene, abscisic acid, jasmonic acid and REDOX signalling (Müller and Munné‐Bosch 2015). ERF1 and ORA59 regulate the ERF branch of JA/ET signalling and serve as integrators between the JA/ET and SA pathways (Lorenzo et al. 2003; Pré et al. 2008).

The LBD transcription factor family includes highly conserved lateral organ boundaries (LOB) domains that play roles in biotic stress by influencing plant lateral organ development or hormone homeostasis (Majer and Hochholdinger 2011; Shuai et al. 2002). At present, LBD has been found and reported to participate in plant disease resistance. The ectopic expression of *AtLBD20* in *Arabidopsis thaliana* inhibits the expression of JA‐regulated defence genes *THI2.1* and *VSP2*, increasing its susceptibility to *Fusarium* (Thatcher et al. 2012). The interaction of *LBD16* with *Meloidogyne* increased the infection rate of plant root‐knot nematodes (Cabrera et al. 2014). After the pathogen *PtoDC3000* enters the host cell, it can synthesise and release auxin to induce the development of lateral roots of the plant through the IAA14‐ARF7/9‐LBD16/18 pathway, thus creating more entry sites for the pathogen (Kong et al. 2020). Studies have also demonstrated that CsLOB1 negatively affects citrus resistance to *Xanthomonas campestris* pv. citri (Xcc). Xcc secretes the main pathogenic factor PthA, which combines with the EBE region of the CsLOB1 promoter to regulate the gene expression related to cell wall remodelling and the plant hormone signalling pathway in citrus, promoting the formation of pustules and the reproduction of Xcc (Long et al. 2021; Zou, Du, et al. 2021).

This study aimed to investigate how the *CsLOB1* from the LBD family regulates citrus responses to *P. citricarpa* using transcriptomic analysis, molecular interaction studies, reverse genetic function verification and biochemical analysis. The results of this study finally revealed the interaction between *CsLOB1* and *CsERF027* and jointly promoted the JA/ET‐dependent ERF1/ORA59 signalling pathway mediated by CsRAP2.3‐CsERF1 cascade transcription, thereby enhancing plants' resistance to *P. citricarpa*. These findings also provide new insights into the mechanism of action of *CsLOB1*, expanding the potential utility of *CsERF027*, *CsRAP2.3* and *CsERF1* as breeding targets for citrus disease resistance.

## 2 Experimental Procedures

### 2.1 Plant, Pathogen Materials and Treatment

The seeds of Wanjincheng ( *Citrus sinensis* , WJC) used in this experiment were collected from the breeding nursery of the Citrus Research Institute, Southwest University, Chongqing. The *CsLOB1* RNAi transgenes WJC plants RB32 and RP30, and the *CsLOB1* overexpression transgenes WJC plants C1 and C4 were all donated by Professor Zou Xiuping (Citrus Research Institute, Southwest University) and planted in pots for open cultivation. *Nicotiana benthamiana* (tobacco) seeds were donated by Professor Zou Xiuping and stored at −4°C. Healthy and disease‐free seeds were soaked and sown in substrate soil before use. The seeds were cultured at 28°C under constant sunlight for 4 weeks. Citrus seedlings, including Eureka, WJC and Tarocco blood orange ( *Citrus X sinensis* ), are purchased from Chongqing Kezheng Flower and Fruit Seedlings Co. Ltd., growing in the open in pots. The leaves and branches of the citrus were collected from the citrus nursery of National Germplasm Resources, Citrus Research Institute, Southwest University. The pathogen of citrus TSD (*P*. *citricarpa*) was isolated from diseased leaves of Eureka, Wanzhou and Chongqing, and was stored in the refrigerator at −40°C in this experiment. The pathogens were cultured on PDA medium for 7–10 days at 20°C before inoculation.

The young citrus shoots with the same maturity were selected to inoculate pathogens, and wounds were made with a soldering iron. The young leaves that did not turn green or the leaves after seed germination were selected for the inoculation test, and the wounds were made with insect needles. Wash the branches and leaves with 75% alcohol once, and then wash them with sterile water 3–5 times. The control group (CK) was inoculated with sterile 5 mm PDA medium (free of pathogens), and the treatment group of each citrus leaf and shoot (Eureka lemon, Tarocco orange and Wanjing orange) was inoculated with 5 mm pathogen medium (containing pathogens). The plants inoculated with *P*. *citricarpa* were placed in sealed trays and cultured in a constant greenhouse at 20°C. Each treatment group received 15 branches or leaves, each with two inoculation points. Four biological replicates were conducted per group. On the fifth day post‐inoculation for branches and the eighth day for leaves, the cross method was used to measure the diameter of the disease spots. Choose healthy, virus‐free WJC saplings with robust growth and fresh young shoots. Spray them with an aqueous solution containing 2 mM SA, 100 μM MeJA and 10 mM ethephon using a fine mist from a small watering can. Apply the solution to three saplings per treatment. The control group should be sprayed with distilled water.

### 2.2 RNA Sequencing and RT‐qPCR Analysis

The pathogen *P. citricarpa* was inoculated on young leaves of Tarocco and Eureka seedlings with insect needles, and blank PDA was inoculated as a control. Leaf samples were collected before treatment (CK_Tco, CK_Ulim) and after treatment for 24 h (WZ1_Tco, WZ1_Ulim), quickly frozen in liquid nitrogen and stored at −80°C for RNA extraction. There were 3 biological replicates in each group. The infection of Tarocco at 0 h and 24 h by *P. citricarpa* was named CK1_Tco and WZ1_Tco, respectively. *P. citricarpa*‐infected Eureka at 0 and 24 h were named CK1_Ulim and WZ1_Ulim, respectively. Leaf samples of each group were extracted with the FastPure Universal Plant Total RNA Isolation Kit, converted into cDNA with All‐In‐One 5X RT MasterMix and stored at −20°C for later use. WZ1_Tco indicates Tarocco inoculation with *P. citricarpa* for 24 h, while WZ1_Ulim denotes Eureka inoculation with *P. citricarpa* for the same duration.

The obtained cDNA was segmented, and the two ends were connected by PCR amplification to obtain a cDNA library for sequencing. The cDNA library was sequenced by the Illumina high‐throughput sequencing platform, and many raw reads were generated. Clean reads were obtained after filtration and compared to the *Citrus sinensis* (L.) Osbeck genome V1.0 in CPDB. Bowtie2 was used to calculate the gene comparison rate (Liu, Wang, et al. 2022). The log_2_ FC > 2 or log_2_ FC < −2 as a threshold, DEG‐seq was used for differentially expressed gene (DEG) analysis and FPKM was calculated according to gene length to determine gene expression level. DESeq2R software was used to analyse the DEGs between each group. KEGG and GO enrichment analysis was performed on the obtained DEGs to search for related genes or metabolic pathways that might be related to the resistance to TSD.

The obtained cDNA was used as a template and diluted 10 times with DEPC water. Then, the primer mixture was configured as the RT‐qPCR primer for each candidate gene according to the ratio of F primer: R primer: H_2_O = 1:1:2. The RT‐qPCR reaction was configured with a 10 μL system according to the cDNA dilution template of 4 μL, a primer mixture of 1 μL and SYBR qPCR Master Mix of 5 μL (Liu, Lv, et al. 2023). The citrus β‐actin was used as the internal reference gene. The reaction procedure was 95°C for 30s, 95°C for 5 s and 60°C for 30 s, a total of 40 cycles. Three biological repetitions and three technical repetitions were set for each sample. The relative gene expression levels in each treatment group were calculated by the 2^‐∆∆CT^ method.

### 2.3 DAB Staining and Determination of H_2_O_2_ and MDA Contents

First, DAB initial dye with a concentration of 1 mg/mL was configured, and the pH was adjusted to 3.0 using 0.2 mM HCl. Add 25 μL (0.05%) Tween 20 and 2.5 mM Na_2_HPO_4_ into the centrifuge tube. The decolorising solution is mixed according to the ratio of ethanol:acetic acid:glycerol = 3:1:1. Leaves of *CsLOB1* transgenic and WT plants inoculated with *P*. *citricarpa* for 2 days were clipped, five leaves in each group and 250 mL DAB staining solution was added. The stain samples were vacuumed for 20 min and placed in a shaker at 28°C, at 80–100 r/min, and the leaves were removed 12 h later. Add decolorising solution into the tube and bleach in a boiling water bath for 15 min. Then, the fresh decolouring solution was replaced and placed at room temperature for 30 min. After H_2_O_2_ in the leaves reacted with DAB, brown patches appeared and photos were taken for the record. Leaf samples of *P*. *citricarpa* infected with *CsLOB1* overexpression, RNAi silencing and WT plants were collected at 0, 2 and 4 days. According to the proportion of 0.1 g sample adding 1 mL of extraction solution, the sample was fully ground with a mortar, and the H_2_O_2_ and malondialdehyde (MDA) content in each treated leaf was determined by the Solarbio kit for hydrogen peroxide (H_2_O_2_) and MDA detection.

### 2.4 Yeast One‐Hybrid (Y1H) Assay

This experiment in Jaspar (https://jaspar.genereg.net/) online software on potential target genes of transcription factors and selection to the promoter sequences of binding sites prediction (Mathelier et al. 2016). The FastPure Plant DNA Isolation Mini Kit was used to extract WJC genomic DNA. The promoter sequences of *CsRAP2.3*, *CsERF1*, *CsPrx15* and *CsPrx64* were amplified with specific primers and constructed into pHis2 vectors (decoys) to form recombinant vectors. The total RNA of the WJC was converted to cDNA using the HiScript II 1st Strand cDNA Synthesis Kit. The coding sequences of *CsLOB1*, *CsRAP2.3* and *CsERF027* (Table S2) were amplified and connected to the PGADT7‐Rec2 vector (prey) to construct a recombinant vector. Then, the self‐activation test group PGADT7‐Rec2 + pHis2‐proRAP2.3/proERF1 and experimental group PGADT7‐Rec2‐*CsLOB1*/*CsRAP2.3*/*CsERF027* + pHis2‐promoter were transferred into Y187 yeast receptor cells (Wang et al. 2023). Three to five transformants were selected and placed on SD/−Leu/−His/−Trp medium containing 3‐AT (OD600 = 0.1/0.01/0.001/0.0001) at 30°C for inverted culture for 3–5 days, and the growth of yeast cells was observed.

### 2.5 Dual‐Luciferase Reporter (Dual‐LUC) Assay

The coding sequences of *CsLOB1*, *CsRAP2.3* and *CsERF027* were constructed into pLG, a plant expression vector with the CaMV 35S promoter. The promoter sequences of *CsRAP2.3* and *CsERF1* were amplified with specific primers (Table S2) and constructed onto the pGreenII 0800 LUC vector. Then the recombinant vector was transferred into Agrobacterium GV3101 competent cells using the liquid nitrogen freeze–thaw method. The Agrobacterium solution containing PLG‐*CSLOB1*, PLG‐*CSRAP2.3*, pGreenII‐promoter recombinant plasmid, pLG and pGreenII‐promoter empty carrier plasmid was mixed 1:1 and transfected into *Nicotiana benthamiana*. Cultured in a constant greenhouse at 28°C for 2–3 days. Tobacco leaves were collected, and the activity of firefly luciferase and Renilla luciferase was tested with the Duo‐Lite Luciferase Assay System kit, and the final values were calculated. Tobacco leaves without injection were the blank control group, pLG empty or recombinant plasmid + pGreenII promoter were the experimental group Firefly and experimental group Renilla, and pLG empty + pGreenII empty were the control group Firefly and control group Renilla.

### 2.6 Yeast One‐Hybrid (Y2H) Assay

In this experiment, *CsLOB1* was constructed into the bait vector PGBKT7, and the candidate gene coding sequence was constructed into the prey vector PGADT7. We took PGBKT7‐CsLOB1 + PGADT7 empty as the self‐activated control group and transferred PGBKT7‐CsLOB1 + PGADT7‐gene CDS together into the Y2HGold yeast competent cell. In this experiment, pGBKT7‐53 + pGADT7‐T was used as a positive control, and pGBKT7‐Lam + pGADT7‐T was used as a negative control. The transformed liquid was cultured inversely in yeast‐screened SD/−Leu/−Trp di‐deficient medium at 30°C for 72 days. Then, 3–5 positive converters were selected and suspended with 20 μL ddH_2_O to OD_600_ = 1. In addition, 5 μL of suspension was absorbed and placed on yeast screening medium containing 3‐AT, SD/−Ade/−Leu/−His/−Trp and SD/−Ade/−Leu/−His/−Trp + X‐gal, and cultured inverted at 30°C for 3–5 days to observe the growth of yeast cells.

### 2.7 Bimolecular Fluorescence Complementation (BiFC) and Subcellular Localisation

The recombinant vectors pSPYENE‐CsLOB1 and pSPYECE‐CSERF027 were constructed by linking the coding sequences of *CsLOB1* and *CsERF027*, respectively. The recombinant vector is then transferred into the Agrobacterium GV3101 competent cell. Agrobacterium solution containing pSPYECE‐CsLOB1 and pSPYENE‐CsERF027 recombinant plasmid was mixed 1:1 to transfect tobacco plants. The tobacco leaves were cultured in a constant greenhouse at 28°C for 2–3 days, and then the fluorescence was observed under a confocal laser microscope. The coding sequences of *CsERF027* and *CsRAPP2.3* were connected to 35S::GFP to construct a recombinant vector and then transferred to Agrobacterium GV3101. A mixture of Agrobacterium containing the target gene and nuclear plasmid bacteria was used to transfect tobacco plants. After 3 days of culture in the absence of light, tobacco leaves were collected and examined for fluorescence using a confocal laser microscope.

### 2.8 Analysis of Transcriptional Activation Activity

The coding sequences of *CsERF027* and *CsRAPP2.3* were connected to pGBKT7 to construct the recombinant vector by referring to the method of Long et al. (Long et al. 2021). In this experiment, PGBKT7‐CSERF027/CsRAPP2.3 + pGADT7 empty was used as the experimental group and pGBKT7 + pGADT7 as the control group, which were transformed into the Y2HGold competent cell of yeast. The transformed yeast cells were plated on SD/−Leu/−Trp medium and cultured inverted at 28°C for 3–5 days. The initial concentration of the bacterial solution was adjusted to 0.1 (OD600) and then diluted 10‐fold and 100‐fold, respectively. Yeast screening SD/−Leu/−His/−Trp triple‐deficiency medium was used for further screening of positive clones, and X‐α‐gal staining was used to detect galactosidase activity. The plate was placed in inversion in an incubator at 28°C for 3 days to observe the growth and blue colour changes of yeast cells.

### 2.9 Virus‐Induced Gene Silencing (VIGS)

Fragments of the target gene's coding sequence, ranging from 300 to 400 base pairs, are selected and inserted into TRV2 vectors to create recombinant plasmids. These plasmids are then introduced into Agrobacterium EHA105 competent cells, which are used to infect citrus seeds. The vector containing TRV1 was mixed 1:1 with the recombinant vector containing either TRV2 empty or TRV2–target gene in Agrobacterium solution and incubated at 28°C for 3 h for seed infection. The seeds of WJC were stripped of its seed coat, and the germination was promoted at 20°C with moist high‐temperature sterilised sand for 7–10 days. After seed germination, seeds with neat and healthy buds were selected and immersed in Agrobacterium suspensions. They were then placed in a vacuum pump under negative pressure for 1 min, and this process was repeated once. The infected seeds were washed with dd H_2_O for 2–3 times and then moved to the seedling tray and cultured at 25°C and 85% humidity. Each treatment involved 20 seeds, repeated 4 times. After 15 to 25 days of citrus seedling growth, three leaves were randomly collected from each replicate group, totalling 12 leaves per treatment. DNA and total RNA were extracted from the leaf tissues and then reverse‐transcribed into cDNA. PCR was used to verify the target fragments of TRV1 and TRV2 on the vector, and RT‐qPCR was used to analyse gene expression levels, and disease resistance of the plant was evaluated.

### 2.10 Transient Gene Overexpression in Citrus

In this study, the target gene sequence was linked to the pLG overexpression vector by homologous recombination to construct a recombinant plasmid. PLG‐target gene vector plasmid is transformed into *Agrobacterium* EHA105 competent cells and transfected into citrus leaves for overexpression of target genes (Li et al. 2024). WJC seeds were stripped of their seed coats and germinated at 20°C with moist, high‐temperature‐sterilised sand for 7–10 days before transplanting to a seedling tray. After the new leaves of the citrus seedlings grow in 15–25 days, gently cut the backs of the citrus seedlings' leaves with a needle. An *Agrobacterium* solution containing the target gene was then injected from the nonleaf vein on the back of the leaf (as a control with pLG empty). Each treatment involved 20 seedlings, repeated 4 times. After incubation in a constant greenhouse at 28°C for 3–5 days, three leaves were randomly collected from each repetition, resulting in a total of 12 leaves per treatment. The DNA and total RNA were extracted from leaf tissues and reverse‐transcribed into cDNA. PCR was used to verify pLG fragments on the vector, and RT‐qPCR was used to analyse gene expression levels.

## 3 Results

### 3.1 Disease Resistance Evaluation and Transcriptome Analysis of Citrus

**FIGURE 1:**
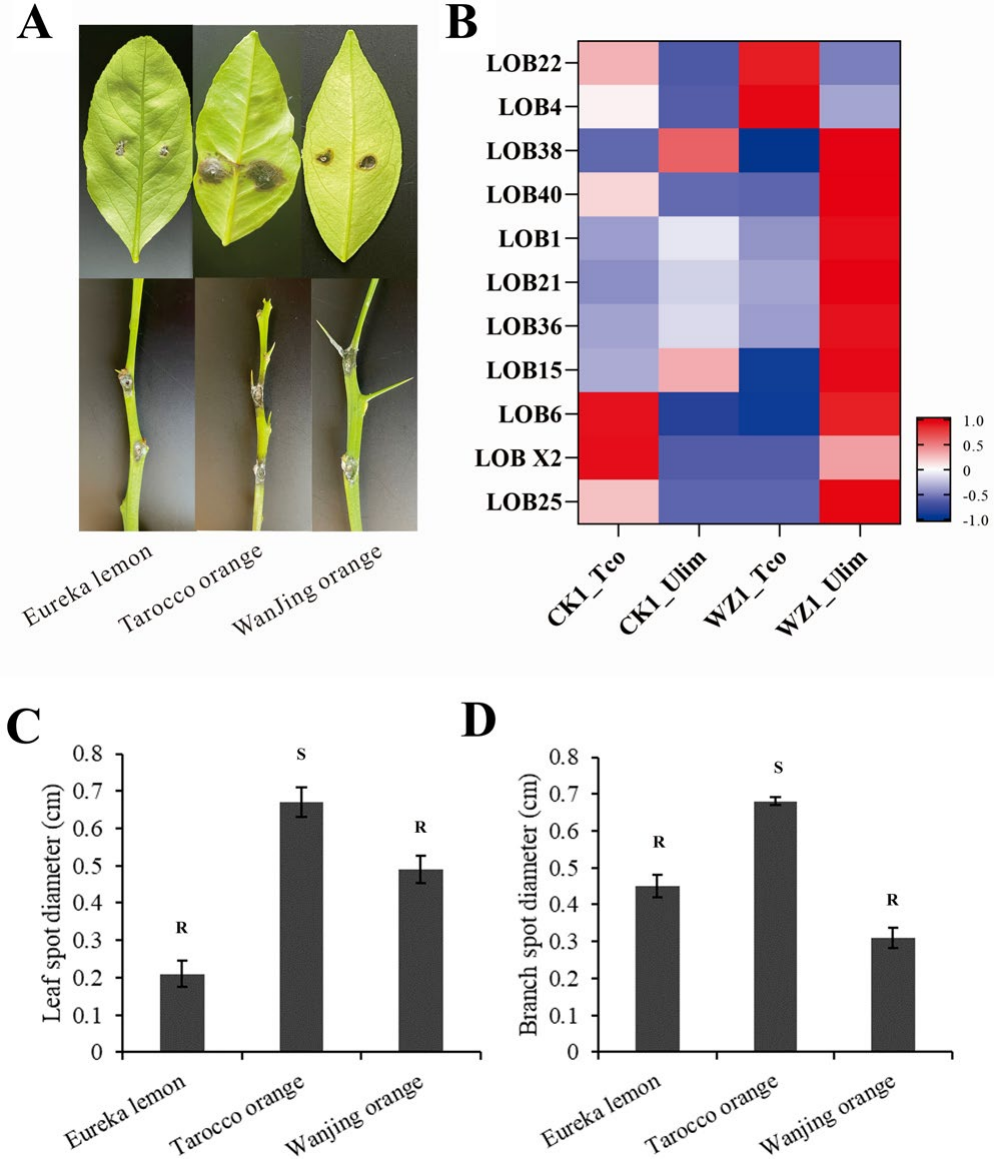
Disease resistance evaluation and transcriptome analysis of citrus cultivars. (A) Diseased spots (Bar = 1 cm) on the leaves and branches of WJC, Tarocco and Eureka after 8 days by *P. citricarpa* infection. (B) The expression changes in LBD transcription factors under different treatments. Different colours in the heat map indicate different levels of gene expression. (C,D) Spot diameter and resistance grade of citrus branches and leaves infected with *P. citricarpa* at 8 days. R indicates resistance grading is resistant, and S indicates resistance grading is susceptible.

After 5 days of infection by *P. citricarpa*, black‐brown fusiform spots appeared at the inoculation sites. The diameters of these spots were 0.45 cm on Eureka, 0.31 cm on WJC and 0.68 cm on Tarocco. After 8 days of infection by *P. citricarpa*, the lesion spot diameters were 0.67 cm for Tarocco, 0.21 cm for Eureka and 0.49 cm for WJC (Figure 1A,C,D). Citrus leaves and branches were graded according to the standard set by Liu, Qiao, et al. (2022). Eureka and WJC were identified as resistant varieties (R) to *P. citricarpa*, while Tarocco was classified as a susceptible variety (S).

RNA‐seq analysis was performed on the leaves of Tarocco and Eureka infected by *P. citricarpa* at 0 and 24 h. The proportion of Q30 was more than 92%, and the GC content was 43.65%–43.72% (Table S1). Correlation analysis results among samples showed that Pearson correlation coefficients were all higher than 0.737 (Figure S1A). The results of hierarchical cluster analysis showed that there were significant differences in the gene expression levels of Tarocco and Eureka leaves infected with *P*. *citricarpa* at 24 h (Figure S1B). Ten genes were randomly selected for RT‐qPCR analysis. The results indicated that the relative gene expression levels from RT‐qPCR aligned with the log_2_FC trends observed in RNA‐seq analysis, confirming the accuracy and reliability of the RNA‐seq data in this experiment (Figure S1C). A total of 5781 DEGs were screened in the comparison group of WZ1_Tco versus WZ1_Ulim, of which 2972 were upregulated and 2809 were downregulated (Figure S1D).

GO and KEGG enrichment analyses were conducted for the differentially expressed genes (DEGs) in WZ1_Tco and WZ1_Ulim (Figure S1E,F). The findings indicate that upregulated DEGs are significantly enriched in pathways related to plant–pathogen interactions, amino sugar and nucleotide sugar metabolism and MAPK signalling (Figure S1G). Down‐regulated differentially expressed genes (DEGs) are significantly enriched in the glycosphingolipid biosynthesis and photosynthesis‐antenna proteins pathways (Figure S1H). Among these, numerous transcription factors exhibit differential expression between WZ1_Tco and WZ1_Ulim, indicating that transcription factors may be involved in the citrus response to TSD infection. Therefore, based on RNA‐seq analysis, 11 LBD transcription factors were screened in this experiment, and their expression levels changed significantly by *P*. *citricarpa* infection (Figure 1B).

### 3.2 Analysis of *CsLOBs* Response to *P. citricarpa* Infection and Hormone Induction

**FIGURE 2:**
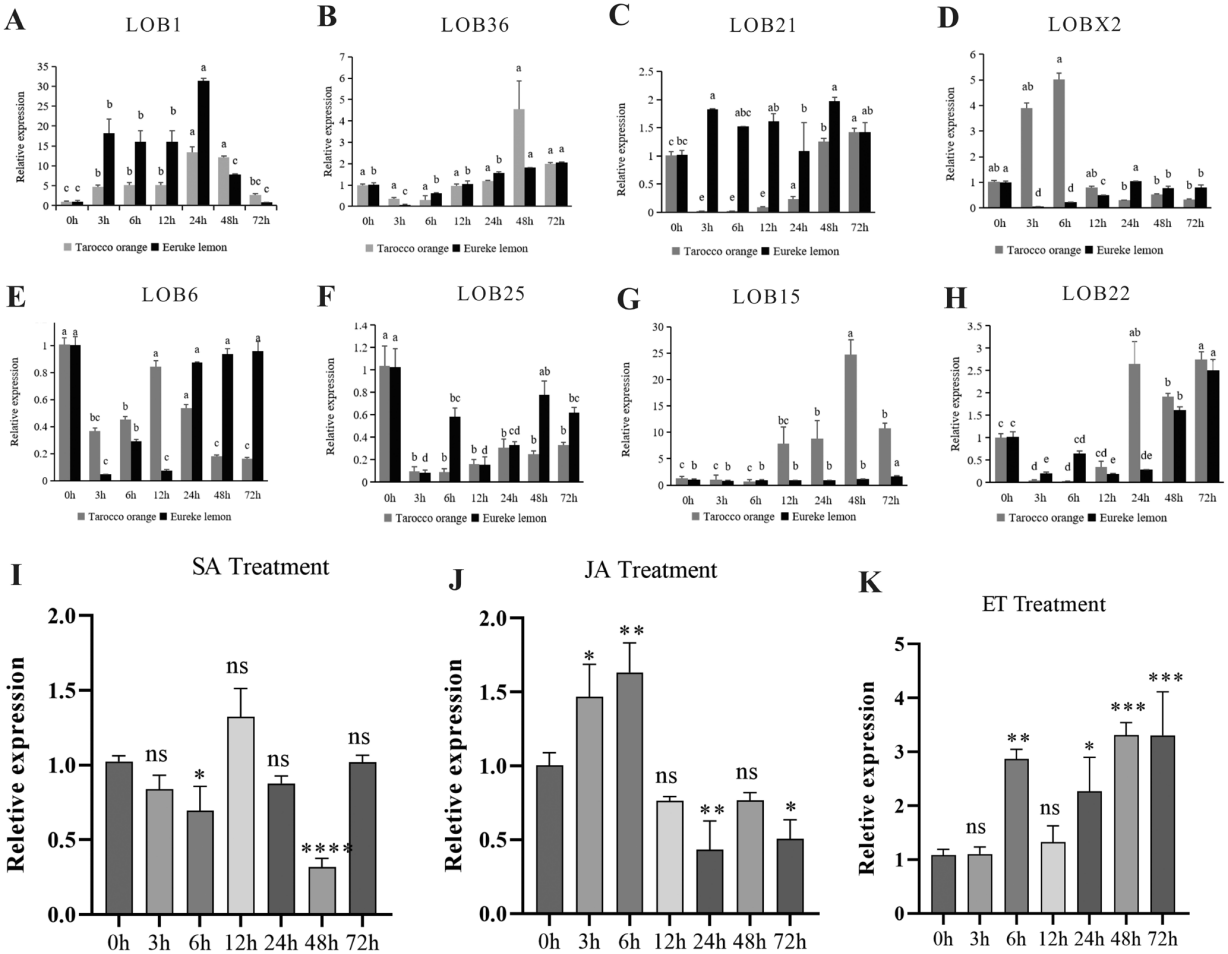
The expression changes of eight candidate LBD family genes in response to *P. citricarpa* infection and *CsLOB1* in response to hormone induction. (A–H) The expression level of LOB1/LOB36/LOB21/LOBX2/LOB6/LOB25/LOB15/LOB22 by *P. citricarpa* infection at 0, 3, 6, 12, 24, 48 and 72 h. Different letters represent significant differences between the two lines. (I–K) The expression level of *CsLOB1* in response to SA/JA/ET treatment at 0, 3, 6, 12, 24, 48 and 72 h. ns: *p* > 0.05, *p < 0.05, **p < 0.01, ***p < 0.001, ****p < 0.0001.

The transcription levels of *LOB1*, *LOB36*, *LOB21*, *LOBX2*, *LOB6*, *LOB25*, *LOB15* and *LOB22* were analysed at 0, 3, 6, 12, 24, 48 and 72 h after *P. citricarpa* infection with Tarocco and Eureka. Among them, the expression level of LOB1 increased with the pathogen infection time, reaching the highest level at 24 h and the lowest level at 72 h (Figure 2A). The expression level of CsLOB1 in Eureka was significantly higher than that in Tarocco. In Eureka, the expression level of LOB36 first decreased and then increased, reaching the highest level at 72 h after infection by *P*. *citricarpa*. However, in Tarocco, the expression level of LOB36 first decreased and then increased, reaching the highest level at 48 h (Figure 2B). In Tarocco and Eureka, the expression levels of *LOB21*, *LOBX2*, *LOB6*, *LOB25* and *LOB22* showed irregular changes and were low (Figures 2C,D,E,F,H). The expression level of *LOB15* increased with the time of infection and reached the maximum at 48 h in Eureka during *P. citricarpa* infection, while *LOB15* was down‐regulated at 3 and 6 h and unregulated at other time periods in Tarocco (Figure 2G). In response to *P*. *citricarpa* infection, the expression level of *LOB1* in disease‐resistant varieties was significantly higher than that in susceptible varieties, while the expression level of *LOB15* in susceptible varieties was significantly higher than that in resistant varieties.

The expression level of *CsLOB1* was analysed after 0, 3, 6, 12, 24, 48 and 72 h of treatment with external spraying of SA, MeJA and ethylene aqueous solution. After SA treatment, the relative expression level of *CsLOB1* was downregulated at 3 and 6 h, upregulated at 12 h, downregulated at 24 and 48 h and returned to the initial level at 72 h (Figure 2H). After exogenous MeJA, the relative expression level of *CsLOB1* was first increased and then decreased (Figure 2I). After ethephon treatment, the relative expression level of *CsLOB1* was up‐regulated at all seven time points (Figure 2J). The above results indicated that *CsLOB1* could respond to the induction of *P*. *citricarpa*, SA, MeJA and ET. In addition, the expression level of *CsLOB1* was upregulated by *P*. *citricarpa*, MeJA and ET treatment, while the expression level of *CsLOB1* was downregulated by SA treatment.

### 3.3 *CsLOB1* Positively Regulates the Resistance of Citrus to *P. Citricarpa*

**FIGURE 3:**
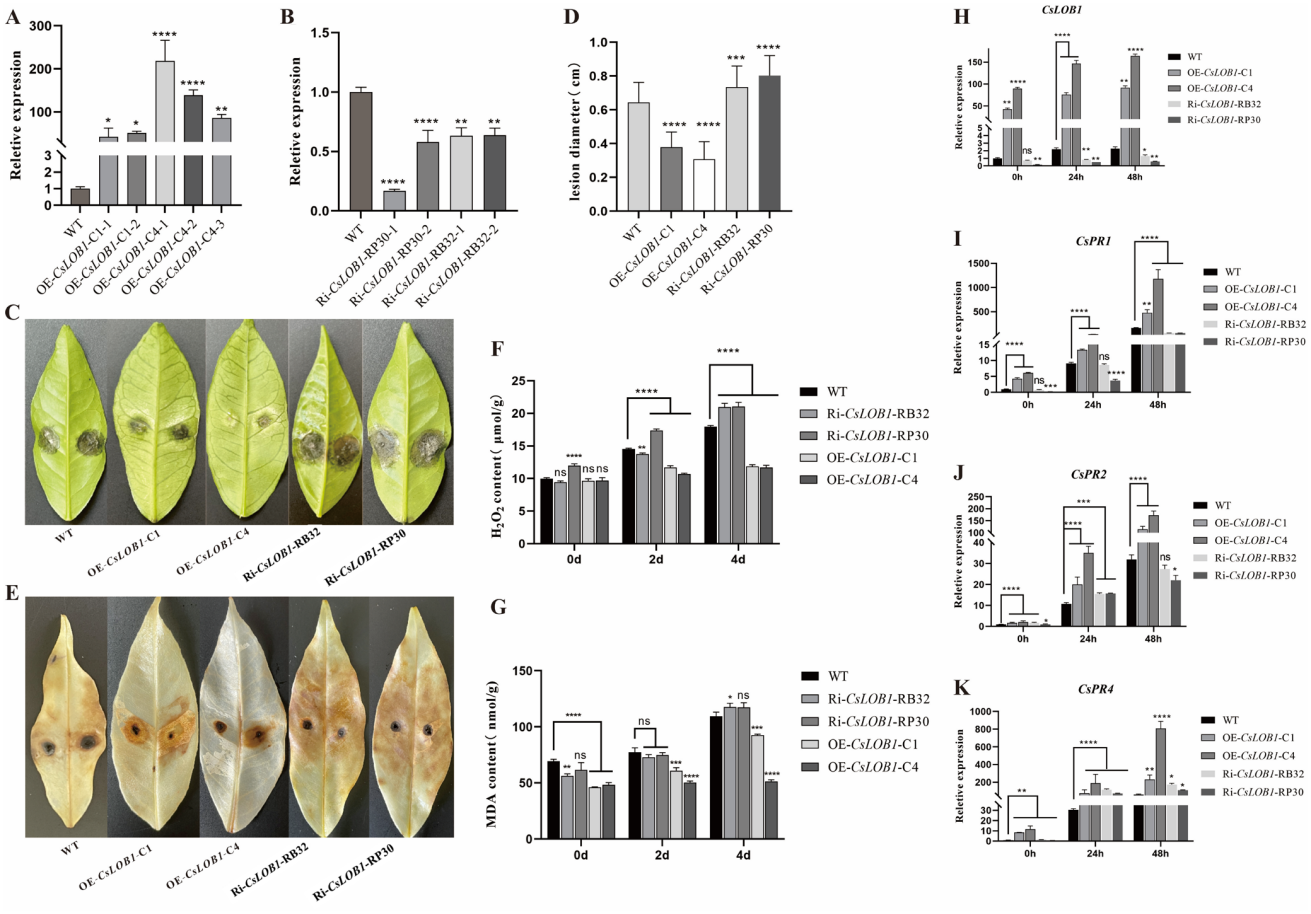
(A) Changes of relative gene expression levels in *CsLOB1* overexpressing transgenic plants. (B) Changes in relative gene expression levels in *CsLOB1* RNAi transgenic plants. (C) Lesions of *CsLOB1* transgenic WJC inoculated with *P. citricarpa* for 8 days. (D) spots diameter of *CsLOB1* transgenic WJC after inoculation with *P. citricarpa* for 8 days. (E) DAB staining results of *CsLOB1* transgenic WJC after inoculation with *P. citricarpa* for 2 days. (F) H_2_O_2_ content in leaves of *CsLOB1* transgenic WJC inoculated with *P. citricarpa* 0, 2 and 4 days. (G) MDA content in leaves of *CsLOB1* transgenic WJC inoculated with *P. citricarpa* 0, 2 and 4 days. (H–K) Relative gene expression levels of *CsLOB1*, *PR1*, *PR2* and *PR4* in *CsLOB1* transgenic WJC inoculated with *P. citricarpa* for 0, 12 and 24 h (ns: *p* > 0.05; *p < 0.05; **p < 0.01; ***p < 0.001; ****p < 0.0001).

Expression levels of *CsLOB1* in wild type (WT), overexpressed transgenic plants C1 and C4, and RNAi transgenic plants RP30 and RB32 were analysed by RT‐qPCR. The results showed that the relative expression level of *CsLOB1* was significantly higher in OE‐*CsLOB1*‐C1 and OE‐*CsLOB1*‐C4 than in WT (Figure 3A). The expression levels of *CsLOB1* in RNAi transgenic plants Ri‐*CsLOB1*‐RP30 and Ri‐*CsLOB1*‐RB32 were significantly lower than those of ET (Figure 3B). After WZ1 inoculation on the leaves of WT, OE‐*CsLOB1*‐C1, OE‐*CsLOB1*‐C4, Ri‐*CsLOB1*‐RP30 and Ri‐*CsLOB1*‐RB32, the results showed that the spot diameter of overexpressed transgenic plants was significantly smaller than that of WT. The incidence of RNAi transgenic plants was more severe than that of wild‐type (WT) plants (Figure 3C,D). The average spot diameters of C1 and C4 decreased by 40.6% and 51.6%, respectively, compared to WT. Compared with WT, the average spot diameter of RB32 (0.73 cm) and RP30 (0.80 cm) increased by 14.0% and 25.0%, respectively (Figure 3D).

The qualitative analysis results of DAB staining in this experiment showed that after inoculation with WZ1, H_2_O_2_ accumulation was significantly higher in Ri‐RB32 and Ri‐RP30, and almost the entire leaf was dyed brown (Figure 3E,F). The second is WT, which is centred on the inoculation site, and the accumulation of H_2_O_2_ spreads from inside to outside. OE‐C1 and OE‐C4 accumulated the least amount of H_2_O_2_, which is lower than WT. H_2_O_2_ only accumulated around the inoculation site, and there were no obvious traces of H_2_O_2_ in the rest of the site (Figure 3E,F). The H_2_O_2_ content in the same plant at different time periods after WZ1 inoculation increased significantly, and reached the maximum at 48 h (Figure 3F). The results of MDA content determination indicated that the MDA levels in each plant rose with extended WZ1 inoculation time, peaking at 4 days postinoculation. Specifically, MDA content in WT, Ri‐RB32 and Ri‐RP30 increased significantly with WZ1 infection duration, whereas MDA content in OE‐C1 and OE‐C4 increased only slightly (Figure 3G).

The gene expression level of *CsLOB1*, *CsPR1*, *CsPR2* and *CsPR4* was analysed after 0, 24 and 48 h of inoculation of the WZ1 strain. After 24 and 48 h of inoculation with WZ1, the expression level of *CsLOB1* in all transgenic plants was significantly up‐regulated and reached the highest level at 48 h (Figure 3H). Among them, the gene upregulation degree in Ri‐RP30 was the largest at 48 h. The relative expression level of OE‐C4 was the highest at 48 h (164.1). After inoculation with WZ1, the expression level of *CsLOB1* in all overexpressed transgenic plants was significantly higher than that in WT and RNAi plants (Figure 3H,J,K). This study also found that WZ1 could significantly induce the expression levels of *CsPR1*, *CsPR2* and *CsPR4*. The expression level of *CsPRs* in overexpressed transgenic plants was significantly higher than that in WT and RNAi plants (Figure 3I). At 24 h of inoculation, gene expression levels of *CsPR2* in RNAi‐RB32 and RP30 were higher than those in WT but significantly lower than OE‐C1 and C4 (Figure 3J). In general, whether citrus was infected by WZ1 or not, the expression level of *CsPR4* in overexpressed transgenic plants was significantly higher than that in WT and RNAi transgenic plants (Figure 3K).

### 3.4 CsLOB1 Binds to the CsRAP2.3 Promoter

**FIGURE 4:**
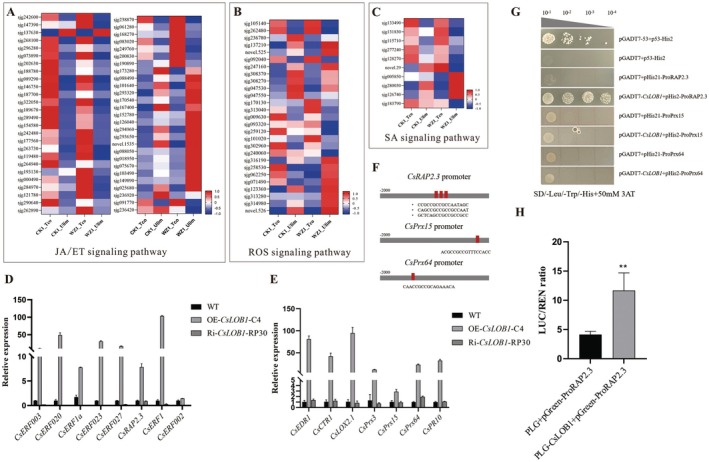
(A) The expression levels of JA/ET signalling pathway‐related genes in four citrus samples. (B) The expression levels of ROS signalling pathway‐related genes in four citrus samples. (C) The expression levels of SA signalling pathway‐related genes in four citrus samples. (D) Differentially expressed genes in *CsLOB1* transgenic WJC (transcription factors). (E) Expression level analysis of differentially expressed genes in CsLOB1 transgenic WJC (functional genes). (F) Prediction of CsLOB1 and downstream gene promoter binding sites. (G) Yeast one‐hybrid system to verify CsLOB1 binding to the CsRAP2.3 promoter. (H) Dual‐Luciferase experiment verified the binding of CsLOB1 to the CsRAP2.3 promoter. **p < 0.01.

In this study, *CsLOB1* is significantly induced by exogenous SA, JA and ET, which has been confirmed. In order to further explore the regulatory pathway of *CsLOB1*‐regulating citrus resistance to target spot disease, detailed analysis was conducted on these related genes (Figure 4A–C). RT‐qPCR analysis of key genes in transgenic plants showed that 15 genes were significantly differentially expressed in *CsLOB1* transgenic plants, including 8 AP2 family transcription factors, *CsERF003*, *CsERF020*, *CsERF1a*, *CsERF023*, *CsERF027*, *CsRAP2.3*, *CsERF1* and *CsERF002* (Figure 4D). It also contained two JA/ET pathway‐related genes (*CsCTR1* and *CsLOX2.1*), three ROS clearance genes (*CsPrx3*, *CsPrx15* and *CsPrx64*) and two SA pathway‐related genes (*CsEDR1* and *CsPR10*) (Figure 4E). Compared with WT, the expression levels of the above genes were significantly up‐regulated in OE‐C4 and significantly down‐regulated in Ri‐RP30. The result indicated that these genes may be induced by *CsLOB1* to participate in the regulation of citrus disease resistance to TSD.

Analysis of transcription factors and gene promoter binding sites revealed three CsLOB1 binding sites on the CsRAP2.3 promoter and one CsLOB1 binding site each on the CsPrx15 and CsPrx64 promoters (Figure 4F). The interaction results of yeast one‐hybrid verification showed that CsLOB1 binds to CsRAP2.3 but does not bind to CsPrx15 and CsPrx64 (Figure 4G). LUC verification results showed that the luciferase value from PLG‐CSLOB1 + pGreenII‐proRAP2.3 was significantly higher than that from pLG empty + pGreenII‐proRAP2.3. CsLOB1 can significantly enhance the transcriptional activity of the CsRAP2.3 promoter (Figure 4H). These results suggested that CsLOB1 could activate the transcription of CsRAP2.3 by regulating the activity of the CsRAP2.3 promoter.

### 3.5 CsRAP2.3 Positively Regulates the Resistance of Citrus to Target Spot Disease

**FIGURE 5:**
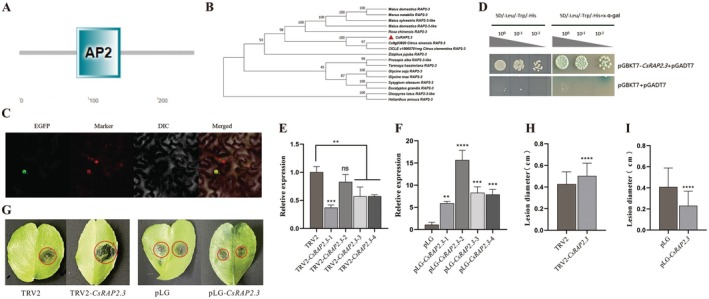
Bioinformatics analysis, subcellular localisation and transcriptional activity analysis of *CsRAP2.3*, *CsRAP2.3* positively regulated citrus target spot disease resistance. (A) Domain prediction of CsRAP2.3. (B) Phylogenetic and evolutionary tree analysis of CsRAP2.3. (C) Subcellular localisation of CsRAP2.3. (D) Transcriptional activity analysis of CsRAP2.3. (E) Gene expression level analysis of *CsRAP2.3*‐overexpressing plants. (F) Analysis of gene expression levels in *CsRAP2.3*‐silenced plants. (G) Changes in leaf spots after 8 days of *CsRAP2.3* overexpression and silenced plants infected by *P. citricarpa*. (H) Changes in the diameter of spots after 8 days of *P. citricarpa* infection with *CsRAP2.3* overexpression plants. (I) Changes in the diameter of spots after 8 days of *P. citricarpa* infection with *CsRAP2.3*‐silenced plants. ns: *p* > 0.05; *p < 0.05; **p < 0.01; ***p < 0.001; ****p < 0.0001.

The *CsRAP2.3* (762 bp) has a typical AP2 domain (Figure 5A), which belongs to the ERF subgroup of the AP2/ERFBP transcription factor family (Nakano et al. 2006). Phylogenetic analysis showed that CsRAP2.3 clustered with *Citrus sinensis* RAP2.3 (Cs9g03820) and *Citrus clementina* RAP2.3 (ciclev10005701mg) (Figure 5B). Subcellular localisation results showed that the fluorescence signal of recombinant protein 35S::CSRAP2.3‐GFP coincided with the fluorescence signal of the nuclear localisation marker on the nucleus, indicating that CsRAP2.3 is a nuclear localisation protein, and the expression signals were also detected on the cell membrane (Figure 5C). In order to detect the transcriptional activation activity of CsRAP2.3 protein, the results showed that the transformed yeast could grow normally in the screening medium SD/−Leu/−Trp/−His and appeared blue in the X‐alpha‐GAL test, indicating that CsRAP2.3 had transcriptional activation activity in yeast cells (Figure 5D).

*CsRAP2.3* silencing plants and overexpressing plants were constructed using citrus TRV‐mediated VIGS transient expression and transient overexpression systems, and pLG or TRV2 empty treatment was used as a control, respectively. The results showed that the gene expression level of *CsRAP2.3* in four *CsRAP2.3*‐silenced plants was significantly lower, which was 0.37, 0.83, 0.57 and 0.57 times compared with WT, respectively (Figure 5E). The gene expression level of *CsRAP2.3* in four overexpressed plants was upregulated, 5.48, 14.14, 7.65 and 7.29 times higher than WT, respectively (Figure 5F). After infecting citrus leaves with *P*. *citricarpa* for 8 days, the incidence of *CsRAP2.3* silent plants was aggravated and spots significantly increased, while the incidence of *CsRAP2.3* overexpression plants was reduced and spots were significantly lower than WT (Figure 5G). The average spot diameter of TRV2 was 0.43 cm, and after *CsRAP2.3* was silenced, the spot diameter was 0.50 cm, which increased by 16.28% (Figure 5H). The average diameter of pLG was 0.41 cm, and after *CsRAP2.3* overexpression, the spots were 0.23 cm, which decreased by 44% (Figure 5I).

### 3.6 CsRAP2.3 Binds to the CsERF1 Promoter to Regulate the Citrus Resistance

**FIGURE 6:**
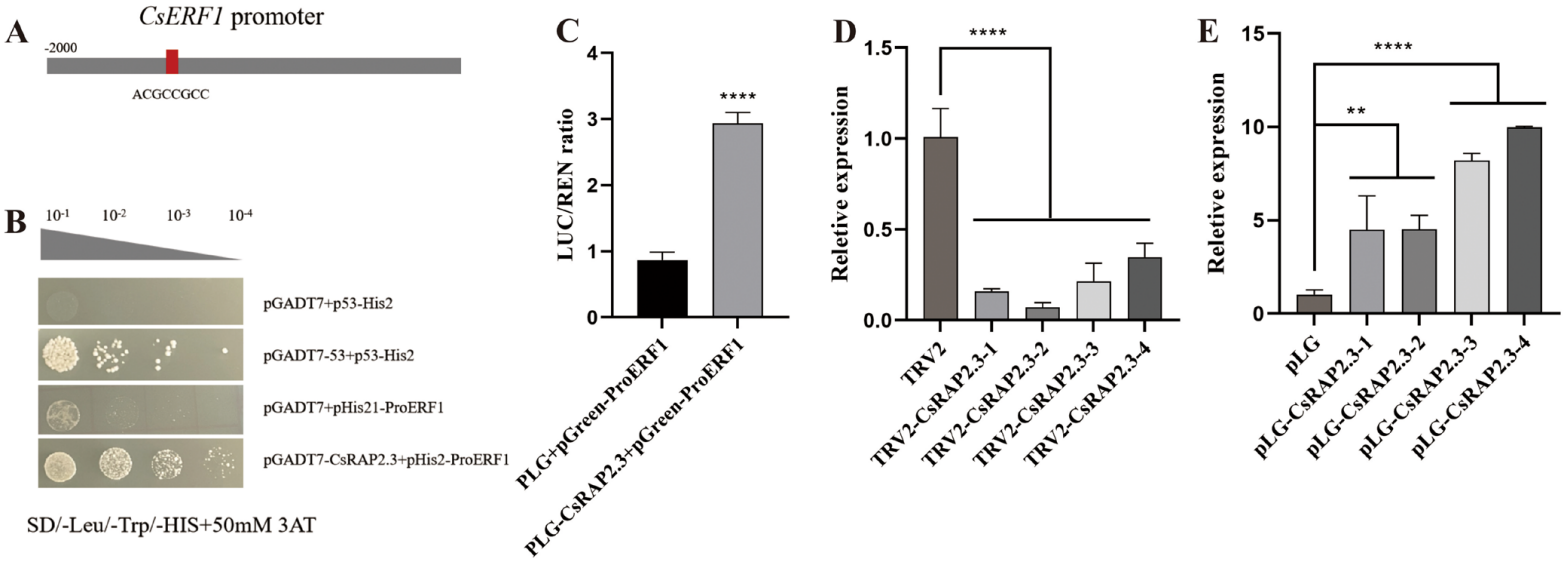
CsRAP2.3 binds to the CsERF1 promoter and regulates its expression. (A) Prediction of the binding site of CsRAP2.3 and the CsERF1 promoter. (B) Yeast one‐hybrid verification of CsRAP2.3 binding to the CsERF1 promoter. (C) The binding of CsRAP2.3 to the CsERF1 promoter was verified by the LUC test. (D) The expression level of CsERF1 in CsRAP2.3‐silenced plants. (E) Expression level of *CsERF1* in *CsRAP2.3‐*overexpressed plants. *p < 0.05; **p < 0.01; ***p < 0.001; ****p < 0.0001.

As the previous experiment found that the expression levels of 15 genes in *CsLOB1* transgenic plants changed significantly, none of the 13 genes except *CsRAP2.3* had a direct effect on *CsLOB1*, indicating that *CsLOB1* had an indirect regulatory effect on these genes. In this experiment, the promoter sequences of 13 genes and the CsRAP2.3 binding site were predicted. We discovered that the CsERF1 promoter contains a CsRAP2.3 binding site (Figure 6A). Yeast one‐hybrid verification results showed that neither the negative control nor the control group (pGADT7 empty + pHis2‐proERF1) grew on TDO medium containing 50 mM 3‐AT. The experimental group (pGADT7‐CsRAP2.3 + pHis2‐ProERF1) grew normally, indicating that CsRAP2.3 could bind to the CsERF1 promoter (Figure 6B).

The results of LUC verification showed that, compared with PLG empty + pGreenII‐proERF1, the activity ratio of luciferase obtained from PLG‐CSrap2.3 + pGreenII‐proERF1 increased significantly. *CsRAP2.3* significantly increased the transcriptional activity of the *CsERF1* promoter (3.39 times) (Figure 6C). This indicated that *CsRAP2.3* could activate the gene transcription of *CsERF1* by regulating the activity of the *CsERF1* promoter. In order to further verify the regulatory effect of CsRAP2.3 on CsERF1, the expression level of *CsERF1* was analysed in *CsRAP2.3‐silenced* and ‐overexpressed plants. The results showed that the expression level of *CsERF1* in *CsRAP2.3*‐silenced plants was significantly lower than that in the control group (TRV2) (Figure 6D), while in *CsRAP2.3*‐overexpressed plants, it was significantly higher than that in the control group (pLG) (Figure 6E).

### 3.7 CsERF1 Positively Regulates the Resistance of Citrus to TSD

**FIGURE 7:**
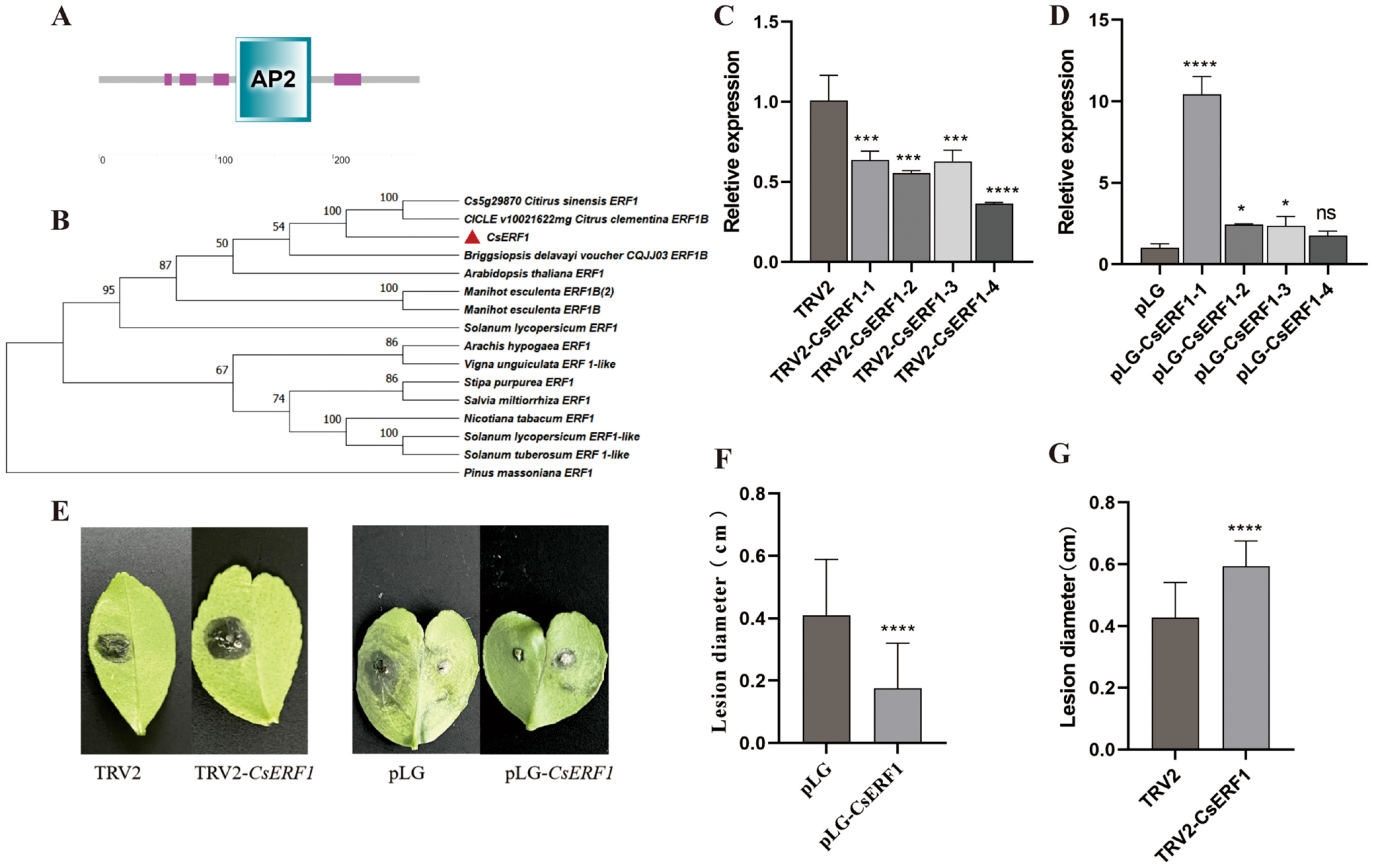
CsERF1 positively regulates the resistance of citrus to target spot disease. (A) CsERF1 domain prediction. (B) Phylogenetic tree analysis of CsERF1. (C) Gene expression level of CsERF1‐silenced plants. (D) Expression level of *CsERF1* in overexpressed plants. (E) Leaf lesions of *P. citricarpa* infected with *CsERF1* overexpression plants and silenced plants after 8 days. (F) The diameter of spots after 8 days of *CsERF1*‐silenced plants infected by *P. citricarpa*. (G) The diameter of spots after 8 days of *CsERF1*‐overexpressed plants infected by *P. citricarpa*. ns: *p* > 0.05; *p < 0.05; **p < 0.01; ***p < 0.001; ****p < 0.0001.

A typical AP2 domain exists on *CsERF1* (Figure 7A), which belongs to the ERF subgroup of the AP2/ERFBP transcription factor family (Nakano et al. 2006). Phylogenetic analysis results show that *CsERF1* is clustered with *Citrus sinensis ERF1* (*Cs5g29870*) and *Citrus clementina* ERF1B (*ciclev10021622mg*) (Figure 7B). In this study, we used the citrus TRV‐mediated VIGS transient expression system and transient overexpression system to construct *CsERF1* silencing plants and overexpressing plants, respectively. PLG and empty TRV2 were used as controls. The results showed that the expression level of *CsERF1* in 4 silenced plants was significantly lower than TRV2 (control) (Figure 7C). Compared with pLG, the expression level of *CsERF1* in four overexpressing plants was significantly upregulated, which was 10.23, 2.40, 2.32 and 1.73 times that of pLG, respectively (Figure 7D). The identification of citrus resistance results showed that, compared with TRV2/pLG, after 8 days of *P*. *citricarpa* infection, the incidence of *CsERF1*‐silenced plants increased significantly, spots enlarged significantly, while the incidence of CsERF1‐overexpressed plants decreased significantly (Figure 7E). In CsERF1‐overexpressed plants, the spot diameter was 0.18 cm, 56.10% smaller than the 0.41 cm diameter observed in *P. citricarpa*‐infected plants with pLG (Figure 7F). In contrast, the spot diameter in *CsERF1*‐silenced plants was 0.59 cm, a 37.21% increase compared to the 0.43 cm diameter seen in *P. citricarpa*‐infected TRV2 plants (Figure 7G).

### 3.8 CsLOB1 Participates in JA/ET Pathway by Regulating CsRAP2.3‐CsERF1 Cascade Transcription

**FIGURE 8:**
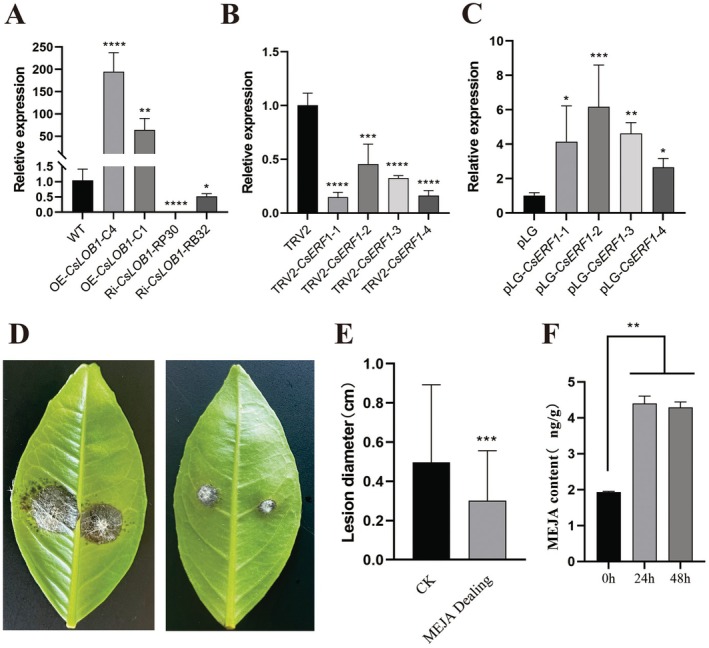
CsLOB1 participates in JA/ET signalling pathway by regulating CsRAP2.3‐CsERF1 cascade transcription. (A) *CsPDF1.2* expression level in *CsLOB1* transgenic plants. (B) *CsPDF1.2* expression level in *CSERF1*‐silenced plants. (C) The expression level of *CsPDF1.2* in *CsERF1*‐overexpressed plants. (D) The leaf lesions of *P. citricarpa*‐infected plants treated with MeJA for 8 days. (E) Average spot diameter of leaves after 8 days of MeJA‐treated plants infected by *P. citricarpa*. (F) MeJA content in leaves after 0, 24 and 48 h of healthy leaves infected by *P. citricarpa*. ns: *p* > 0.05; *p < 0.05; **p < 0.01; ***p < 0.001; ****p < 0.0001.

In order to confirm whether *CsLOB1* regulates the ERF/ORA59 branch in the citrus JA/ET signalling pathway by regulating CSRAP2.3‐CSERF1. In this study, *CsPDF1.2* expression levels in overexpressed and silenced plants of *CsLOB1* and *CsERF1* were detected. The results showed that *CsPDF1.2* was significantly induced in both *CsLOB1‐* and *CsERF1*‐overexpressed plants (Figure 8A,C). However, the expression of *CsPDF1.2* was inhibited (significantly down‐regulated) in *CsLOB1‐* and *CsERF1*‐silenced plants (Figure 8A,B). In this experiment, leaves were inoculated with WZ1 following exogenous MeJA application to assess if MeJA treatment could enhance citrus plants' resistance to TSD. The results indicated that the disease severity in plants treated with MeJA (0.30 cm) was significantly lower than in the control group (CK, 0.50 cm), with the average spot diameter decreasing by 40% (Figure 8D,E). In addition, the MeJA levels of WJC leaves were also measured at different time points of WZ1 inoculation. The results showed that before inoculation with WZ1, the MeJA content in WJC leaves was 1.93 ng/g, and after inoculation for 24 and 48 h, the MeJA content was 4.4 and 4.3 ng/g, increasing by 127.98% and 122.79%, respectively. *P. citricarpa* can significantly induce the increase of MeJA content in WJC leaves (Figure 8F). These results indicated that exogenous MeJA treatment improved the resistance of citrus to *P. citricarpa*.

### 3.9 CsLOB1 Interacts With Transcription Factor CsERF027

**FIGURE 9:**
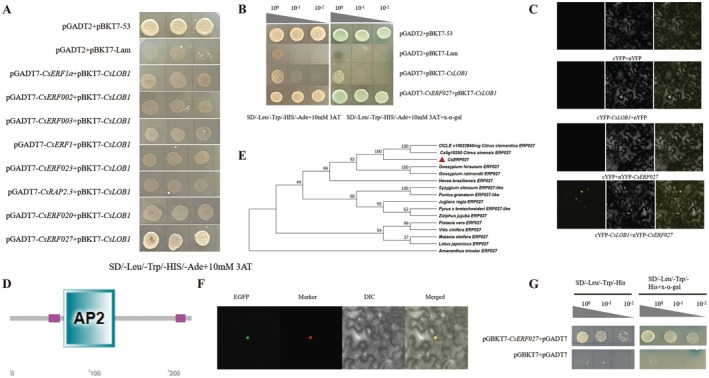
Analysis of interaction between CsLOB1 and transcription factor CsERF027. (A) Yeast two‐hybrid experiment of CsLOB1 with eight transcription factors. (B) Yeast two‐hybrid point‐to‐point verification of CsLOB1 interacting with CsERF027. (C) Interaction between CsLOB1 and CsERF027 verified by BiFC experiment. (D) CsERF027 domain prediction. (E) Phylogenetic tree analysis of CsERF027. (F) Subcellular localisation of CsERF027. (G) Transcriptional activity analysis of CsERF027.

We identified eight differentially expressed AP2 family transcription factors at an earlier stage. Yeast two‐hybrid interaction screening of these eight genes further elucidated the pathway through which *CsLOB1* regulates TSD resistance in citrus. The results showed that CsERF027 interacts with CsLOB1, while the remaining seven AP2 family members do not interact with CsLOB1 (Figure 9A,B). BiFC validation results indicated that pSPYECE‐*CsLOB1* combined with pSPYENE‐*CsERF027* exhibited yellow fluorescence in the nucleus under confocal microscopy, while no fluorescence was observed in the other groups (Figure 9C). These results indicate that the proteins CsLOB1 and CsERF027 are colocalised in the nucleus. A typical AP2 domain exists on CsERF027 (Figure 9D), which belongs to the ERF subgroup of the AP2/ERFBP transcription factor family (Pré et al. 2008; Kim, Jang, and Park 2019). Phylogenetic analysis showed that *CsERF027* was clustered with *Citrus sinensis ERF27* (*Cs5g10250*) and *Citrus clementina ERF27* (*ciclev10023840*) with 100% similarity (Figure 9E). In this experiment, the fluorescence signal of the recombinant protein 35S::CsERF027‐GFP matched that of the nuclear localisation marker, suggesting that CsERF027 is a nuclear‐localised protein (Figure 8F). In order to detect the transcriptional activation activity of the CsERF027 protein, the results showed that the yeast transformed to express CsERF027 grew normally in the screening medium SD‐Leu‐His‐Trp and appeared blue in the X‐alpha‐Gal test (Figure 9G). These results indicate that CsERF027 has transcriptional activation activity in yeast cells.

### 3.10 CsERF027 Regulates Citrus Resistance and Binds to the CsRAP2.3 Promoter

**FIGURE 10:**
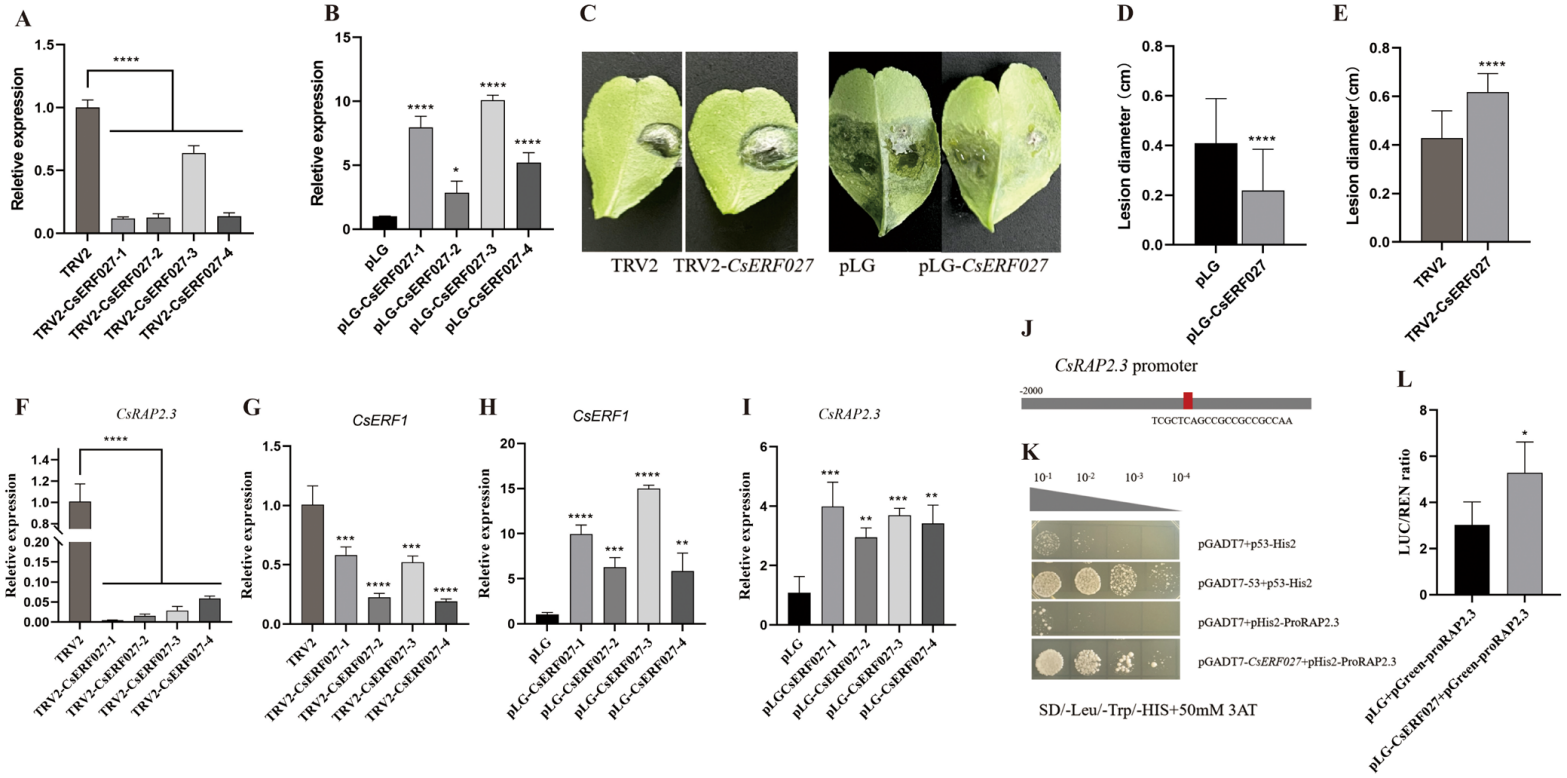
CsERF027 positively regulates TSD resistance in citrus and binds to the CsRAP2.3 promoter. (A) The gene expression level of CsERF027‐silenced plants. (B) The gene expression level of *CsERF027*‐overexpressed plants. (C) Leaf lesions of *P. citricarpa* infected with *CsERF027*‐overexpressed plants and silenced plants after 8 days. (D) Spots diameter of *P. citricarpa* infected *CsERF027*‐silenced plants for 8 days. (E) Spots diameter of *P. citricarpa* infected with *CsERF027*‐overexpressed plants for 8 days. (C) BiFC experiment verified the interaction between CsLOB1 and CsERF027. (D) CsERF027 domain prediction. (H) Phylogenetic tree analysis of CsERF027. (F) The expression level of *CsRAP2.3* in *CsERF027*‐silenced plants. (G) The expression level of *CsERF1* in *CSERF027*‐silenced plants. (H) The expression level of *CsRAP2.3* in *CSERF027*‐overexpressed plants. (I) The expression level of *CsERF1* in *CSERF027*‐overexpressed plants. (J) Prediction of the promoter binding site of *CsERF027* and *CsRAP2.3*. (K) Yeast one‐hybrid experiment verified CsERF027 binding with CsRAP2.3 promoter. (L) LUC experiment verified CsERF027 binding with CsRAP2.3 promoter.

In this study, silencing plants and overexpressing plants of *CsERF027* were constructed, respectively, and TRV2/pLG was used as a control. The results showed that the gene expression levels of the four *CsERF027*‐silenced plants were significantly lower than that of TRV2, which were 0.12, 0.12, 0.64 and 0.14 times that of TRV2, respectively (Figure 10A). The gene expression levels of four overexpressed *CsERF027* plants were 7.97, 2.84, 10.09 and 5.22 times that of pLG, respectively (Figure 10B). After infecting the leaves of plants with *P*. *citricarpa*, the incidence of *CSERF027*‐silenced plants increased, while the incidence of overexpressed plants decreased significantly (Figure 10C). Compared with the spot diameter of 0.43 cm after 8 days of TRV2 infection by *P*. *citricarpa*, the TRV2‐CSERF027 increased by 44.19% (0.62 cm) (Figure 10D). The spot diameter of *P*. *citricarpa*‐infected pLG was 0.41 cm, while the spot diameter of pLG‐CsERF027 was reduced by 43.90% to 0.23 cm (Figure 10E).

**FIGURE 11:**
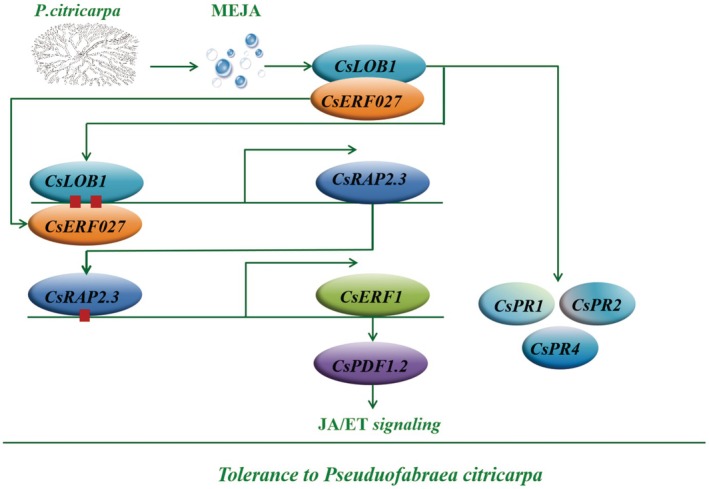
Interaction model of *CsLOB1* regulatory network in response to *P. citricarpa* infection in citrus plants.

In order to further explore the relationship between *CsERF027* and *CsRAP2.3* or *CsERF1*, the results showed that the expression levels of *CsRAP2.3* and *CsERF1* were significantly lower in TRV2‐CSERF027 than in TRV2 (Figure 10F,G). The expression level of PLG‐CSERF027 was significantly higher than pLG (Figure 10H,I). These results indicated that the expression levels of *CsRAP2.3* and *CsERF1* were regulated by *CsERF027*. The direct regulatory effect of *CsERF027* on *CsRAP2.3*, *CsERF1* and *CsLOB1* remains unknown. Consequently, the experiment aimed to predict the *CsERF027* binding sites within the promoters of these genes. The results indicated that the CsERF027 binding site was present solely on the promoter of CsRAP2.3, with a score of 0.89 (Figure 10J). Yeast one‐hybrid verification revealed that neither the negative control nor the control group (pGADT7 empty + pHis2‐proRAP2.3) grew on TDO medium containing 50 mM 3‐AT. The experimental group (pGADT7‐CsERF027 + pHis2‐proRAP2.3) could grow normally (Figure 10K,L). These results suggest that CsERF027 can bind to the promoter of CsRAP2.3 (Figure 11).

## 4 Discussion

Target spot disease (TSD) is an important disease in citrus that threatens citrus production and other fruit industries. According to field monitoring reports, TSD occurred in *Citrus reticulata* ‘Unshi’, *Citrus reticulata* ‘Ponka’, *Citrus japonica* Thunb., *Citrus sinensis* ‘Dahong Chen’, *Citrus erythrosa* Yu. Tanaka, Citrus × limon ‘Eurek’, Newhall navel orange, etc. (Zhan et al. 2021). The chamber disease resistance test results showed that the sensitivity of different varieties of citrus to TSD was significantly different (Liu, Wang, et al. 2022). In Chongqing's citrus‐producing regions, Eureka, WJC and Tarocco blood oranges are the primary varieties, known for their extensive cultivation history and significant planting areas. During the initial phase of this study in 2022, 83 citrus germplasm resources were assessed for their tolerance to TSD. The results showed that Eureka and WJC were resistant varieties and Tarocco was a sensitive variety. Therefore, these citrus varieties were used in this study to provide new insights for the control and resistance breeding of citrus TSD.

Based on RNA‐seq and RT‐qPCR analysis of citrus, we found that the expression level of *CsLOB1* in citrus was significantly upregulated, particularly in Eureka compared to Tarocco. These findings suggest that *CsLOB1* may play a role in enhancing citrus resistance to TSD. Conversely, the expression level of *CsLOB15* was significantly higher in Tarocco than in Eureka, indicating that *CsLOB15* might be associated with increased susceptibility to TSD in citrus. The LATERAL ORGAN BOUNDARIES DOMAIN (LBD) family genes, unique to plants, play a crucial role in the development, secondary growth, regeneration and synthesis of secondary metabolites in lateral organs such as roots, branches and leaves (Gombos et al. 2017; Zhang et al. 2020). At the same time, the LBD family genes also respond to biotic stress and play an important role in regulating plant–pathogen interactions. For example, the expression of *AtLBD20* in *Arabidopsis thaliana* increases susceptibility to *Fusarium* sp. (Thatcher et al. 2012).

The interaction between *LBD16* and *Meloidogyne increased* the infection rate of root‐knot nematode in plants (Cabrera et al. 2014). The pathogen *PtoDC3000* induces lateral root development through the IAA14‐ARF7/9‐LBD16/18 pathway, thereby creating more entry sites for the pathogen (Kong et al. 2020). By binding to PthA effectors in the EBE region, *CsLOB1* regulates genes related to cell wall and plant hormone signalling pathways to increase susceptibility to citrus canker (Long et al. 2021; Zou, Du, et al. 2021). A previous study indicated that CsLOB1 might promote cell proliferation via cell wall remodelling and plant hormone signalling pathways, thus increasing citrus susceptibility to canker pathogens (Zou, Du, et al. 2021). However, its role in regulating fungal diseases in citrus has not been documented. This study reveals that CsLOB1 may play a part in the resistance response of citrus to TSD. In *CsLOB1*‐overexpressed transgenic plants, TSD plaque diameter significantly decreased, while the diameter in RNAi plants significantly expanded. The results showed that overexpression of *CsLOB1* enhanced the TSD resistance of citrus, while silencing of *CsLOB1* reduced the resistance, indicating that *CsLOB1* was a positive regulatory factor that participated in the TSD resistance of citrus.

When plant tissues are stressed by diseases, they activate the expression of related genes and accumulate various toxic substances. In this experiment, the result of DBA staining and H_2_O_2_ and MDA determination showed that after pathogen inoculation, excessive H_2_O_2_ and MDA were accumulated in WT and RNAi‐CsLOB1 plants, while they were significantly reduced in *CsLOB1*‐overexpressed plants. The burst of ROS is an important physiological response in the plant local defence response when pathogenic microorganisms invade plants. High concentrations of ROS and MDA have toxic effects on plant cells (Khan et al. 2022). The findings further demonstrated that *CsLOB1* aids in maintaining ROS homeostasis in plants during TSD infection, thereby reducing cellular damage and enhancing citrus resistance (Mahavihakanont et al. 2012). Additionally, PR proteins play a crucial role in plant defence responses and exhibit antibacterial activity against pathogen infections. In this study, we found that *CsPR1*, *CsPR2* and *CsPR4* were related to the resistance response of citrus and were marker genes of systemic acquired resistance of citrus (Qiu et al. 2020; Zou, Du, et al. 2021). The expression levels of *CsPR1*, *CsPR2* and *CsPR4* in *CsLOB1*‐overexpressed plants were significantly higher than in WT and RNAi‐CsLOB1 plants. These results indicated that *CsLOB1* overexpression in citrus could improve plant defence ability, and *CsLOB1* may directly or indirectly regulate *CsPR1*, *CsPR2* and *CsPR4*.

Previous studies have reported that the LBD transcription factor family can participate in biotic and abiotic stress by regulating hormone homeostasis. Important signalling molecules related to plant defence regulate disease resistance and plant information transmission networks, including Ca^2+^, ROS, SA, JA, ET, NO and heterotrimeric G (Liu et al. 2020; Wang, Zhang, et al. 2021). For example, the ectopic expression of *AtLBD20* inhibits the expression of JA‐related genes *THI2.1* and *VSP2*, making plants more susceptible to *Fusarium* wilt (Thatcher et al. 2012). In this study, the DEGs related to JA/ET, ROS scavengers and the SA pathway were all affected by *CsLOB1*, thus regulating the resistance of citrus to TSD. Among them, *RAP2.3*, which belongs to the ERF family, can participate in physiological processes such as hypoxia response (Papdi et al. 2015), heat shock response (Ogawa et al. 2005), biological stress response (Kim, Jang, and Park 2019), cold‐induced response and waterlogging stress in plant cells (Li et al. 2023; Shi et al. 2021). *RAP2.3* is the target of ET receptor EIN3, and the transcriptional activity of *RAP2.3* is regulated by ET signal transduction (Kim, Jang, and Park 2019; Chang et al. 2013). This study found that *CsRAP2.3* is the downstream target gene of *CsLOB1*, and *CsLOB1* directly binds to the *CsRAP2.3* promoter to regulate the expression level of *CsRAP2.3*. At the same time, *CsRAP2.3*, as a transcription factor of the AP2 family, is involved in regulating the resistance of citrus to TSD, which is a positive regulator of citrus TSD. These results suggest that *CsLOB1* may affect hormone‐related pathways by regulating *CsRAP2.3*, thereby regulating the disease resistance of citrus to TSD.

The complex network of hormone signalling pathways in plants plays an important role in plant disease resistance. Previous studies have shown that exogenous hormones can regulate the expression of LOB (Majer and Hochholdinger 2011; Zou, Du, et al. 2021). In plants, JA is an important hormone in response to abiotic or biotic stress (especially saprophytic bacteria and phytophagous insects), which is an important signalling pathway in plant disease resistance, including the ERF/ORA59 signalling pathway. Among them, *ERF1* is a key gene involved in the regulation of defence response in the JA/ET pathway and can regulate the ERF/ORA59 signalling branch of the JA/ET pathway (Lorenzo et al. 2003). *PDF1.2* is a downstream marker gene of the ERF/ORA59 signalling pathway, and the expression of *PDF1.2* upregulation is a marker for the activation of the ERF/ORA59 signalling pathway (Brown et al. 2003). In this study, we detected CsPDF1.2 gene expression levels in *CsLOB1‐* and *CsERF1*‐overexpressed and ‐silenced plants. In line with previous research, the yeast one‐hybrid system and dual‐luciferase assays in this study confirmed that ERF1, a downstream gene of *CsRAP2.3* in citrus, is regulated by *CsRAP2.3* (Zhu et al. 2014). Based on previous studies, this experiment proves that *CsLOB1* promotes the expression of *CsERF1* and activates the ERF/ORA59 branch of JA/ET signalling pathway through positive regulation of *CsRAP2.3* (Yang et al. 2021). This experiment has confirmed that the expression level of *CsPDF1.2* in *CsLOB1* and *CsERF1* transgenic plants is significantly regulated by *CsLOB1* and *CsERF1*. Exogenous MeJA treatment also significantly improved the resistance of citrus to TSD. These results suggest that *CsLOB1* activates the ERF/ORA59 branch of the JA/ET signalling by regulating the CsRAP2.3–CsERF1 pathway in citrus, thereby improving the citrus resistance.

The study has shown that most regulatory proteins can physically interact with other proteins to form complexes involved in plant stress defence, and their interactions with transcription factors can enhance binding to downstream DNA (Singh et al. 2002). A previous study has found that PpERF98‐1/2 and PpERF1‐1/2 proteins can form heterodimers with themselves or each other to enhance the sensitivity of peach trees to *L. theobromae* (Zhang et al. 2023). In this study, we also found that CsLOB1 can interact with CsERF27 to form heterodimers. As a transcription factor, CsERF27 is involved in the positive regulation of citrus resistance to TSD, which can regulate the expression of CsLOB1 downstream genes *CsERF27* and *CsRAP2.3*. As a member of the LBD family, *CsLOB1* has a typical LOB domain with a typical CX2CX6CX3C‐like zinc finger structure, which is related to the binding of CsLOB1 and downstream DNA (Majer and Hochholdinger 2011). In the C‐terminal of CsLOB1, there exists LX6X3LX6L (a leucine‐like zipper motif), which can interact with other transcription factors to form heterodimers and jointly participate in the regulation of related metabolic pathways (Berckmans et al. 2011; Shuai et al. 2002). In this yeast hybridisation experiment, we verified the binding relationship between CsERF27 and the CsRAP2.3 promoter, indicating that *CsERF27* regulates the resistance of citrus to TSD through the CSRAP2.3‐CSERF1 pathway. However, the interaction of CsLOB1 with CsERF27 and its enhanced transcriptional regulation of the CSRAP2.3‐CSERF1‐mediated JA/ET pathway remains to be further studied.

## 5 Conclusion

In this study, exogenous MeJA, ethephon and the infection of *P. citricarpa* could significantly increase the expression level of *CsLOB1* in citrus. Exogenous application of MeJA could improve the citrus resistance to TSD, and the infection of *P. citricarpa* significantly increased the content of MeJA in WJC. This experiment confirmed that CsLOB1 acts as a positive regulator of citrus defence against TSD by assessing the disease resistance of CsLOB1 transgenic citrus plants and measuring relevant indices. Overexpression of *CsLOB1* significantly increased the expression levels of systemically acquired resistance marker genes *CsPR1*, *CsPR2* and *CsPR4* in citrus. By binding to the promoter of *CsRAP2.3*, *CsLOB1* targeted the CSRAP2.3‐CSERF1 cascade transcription and activated the marker gene *CsPDF1.2* in the JA/ET signalling pathway, thus activating the ERF/ORA59 signalling branch. This experiment confirmed that *CsLOB1* acts as a positive regulator of citrus defence against TSD by assessing the disease resistance of *CsLOB1* transgenic citrus plants and measuring relevant indices.

## Author Contributions

J.H. designed the experiments. F.L., S.L. and J.H. performed the experiments. S.L., F.L., and S.L. analyze data and wrote the paper. X.Q., L.C., Y.Z., and X.Z. analyze data and review the paper.

## Conflicts of Interest

The authors declare no conflicts of interest.

## Supporting information


**Figure S1:** Correlation analysis and enrichment pathway analysis of RNA‐Seq data. (A) Pearson correlation coefficient. (B) Cluster results for four samples. (C) Reliability results of RT‐qPCR‐verified RNA‐Seq data. (D) Differentially expressed gene analysis of WZ1_Tco VS WZ1_Ulim. (E) GO enrichment analysis of upregulated genes in WZ1_Tco VS WZ1_Ulim. (F) GO enrichment analysis of downregulated genes in WZ1_Tco VS WZ1_Ulim. (G) KEGG enrichment analysis of upregulated gene expression in WZ1_Tco VS WZ1_Ulim. (F) KEGG enrichment analysis of downregulated gene expression in WZ1_Tco VS WZ1_Ulim.

**Table S1.** RNA‐Seq data output quality.
**Table S2.** All list primers used in this study.

## Data Availability

The data that support the findings of this study are available on request from the corresponding author. The data are not publicly available due to privacy or ethical restrictions.

## References

[pbi70306-bib-0001] Berckmans, B. , V. Vassileva , S. P. Schmid , et al. 2011. “Auxin‐Dependent Cell Cycle Reactivation Through Transcriptional Regulation of Arabidopsis E2Fa by Lateral Organ Boundary Proteins.” Plant Cell 23, no. 10: 3671–3683. 10.1105/tpc.111.088377. 22003076 PMC3229142

[pbi70306-bib-0002] Berrocal‐Lobo, M. , A. Molina , and R. Solano . 2002. “Constitutive Expression of ETHYLENE‐RESPONSE‐FACTOR1 in Arabidopsis Confers Resistance to Several Necrotrophic Fungi.” Plant Journal 29, no. 1: 23–32. 10.1046/j.1365-313x.2002.01191.x. 12060224

[pbi70306-bib-0003] Brown, R. L. , K. Kazan , K. C. McGrath , D. J. Maclean , and J. M. Manners . 2003. “A Role for the GCC‐Box in Jasmonate‐Mediated Activation of the PDF1.2 Gene of Arabidopsis.” Plant Physiology 132, no. 2: 1020–1032. 10.1104/pp.102.017814. 12805630 PMC167040

[pbi70306-bib-0004] Cabrera, J. , F. E. Díaz‐Manzano , M. Sanchez , et al. 2014. “A Role for LATERAL ORGAN BOUNDARIES‐DOMAIN 16 During the Interaction Arabidopsis‐Meloidogyne spp. Provides a Molecular Link Between Lateral Root and Root‐Knot Nematode Feeding Site Development.” New Phytologist 203, no. 2: 632–645. 10.1111/nph.12826. 24803293

[pbi70306-bib-0005] Chang, K. N. , S. Zhong , M. T. Weirauch , et al. 2013. “Temporal Transcriptional Response to Ethylene Gas Drives Growth Hormone Cross‐Regulation in Arabidopsis.” 2: e00675. 10.7554/eLife.00675. PMC3679525 23795294

[pbi70306-bib-0006] Chen, C. , G. J. Verkley , G. Sun , J. Z. Groenewald , and P. W. Crous . 2016. “Redefining Common Endophytes and Plant Pathogens in Neofabraea, Pezicula, and Related Genera.” Fungal Biology 120, no. 11: 1291–1322. 10.1016/j.funbio.2015.09.013. 27742091

[pbi70306-bib-0007] Chen, Q. , Y. H. Xu , J. H. He , and Y. H. Yang . 2022. “Establishment of an Identification Method of Citrus Resistance to Target Spot.” Journal of Fruit Science 39, no. 2: 295–301. 10.13925/j.cnki.gsxb.20210420.

[pbi70306-bib-0008] Chen, Q. , W. J. Zhang , J. H. He , S. L. Xu , and J. W. Guo . 2023. “Symptoms and Occurrence Regularity of Citrus Target Spot in the Field and the Resistance Determination of 47 Citrus Varieties.” Journal of Fruit Science 40, no. 8: 1675–1691. 10.13925/j.cnki.gsxb.20220564.

[pbi70306-bib-0009] Dombrecht, B. , G. P. Xue , S. J. Sprague , et al. 2007. “MYC2 Differentially Modulates Diverse Jasmonate‐Dependent Functions in Arabidopsis.” Plant Cell 19, no. 7: 2225–2245. 10.1105/tpc.106.048017. 17616737 PMC1955694

[pbi70306-bib-0010] Gfeller, A. , L. Dubugnon , R. Liechti , and E. E. Farmer . 2010. “Jasmonate Biochemical Pathway.” Science Signaling 3, no. 109: cm3. 10.1126/scisignal.3109cm3. 20159849

[pbi70306-bib-0011] Gombos, M. , Z. Zombori , M. Szécsényi , G. Sándor , H. Kovács , and J. Györgyey . 2017. “Characterization of the LBD Gene Family in Brachypodium: A Phylogenetic and Transcriptional Study.” Plant Cell 36: 61–79. 10.1007/s00299-016-2057-0. 27686461

[pbi70306-bib-0012] Halim, V. A. , S. Altmann , D. Ellinger , et al. 2009. “PAMP‐Induced Defense Responses in Potato Require Both Salicylic Acid and Jasmonic Acid.” Plant Journal 57, no. 2: 230–242. 10.1111/j.1365-313X.2008.03688.x. 18801014

[pbi70306-bib-0013] Khan, R. , X. Ma , Q. Hussain , et al. 2022. “Application of 2,4‐Epibrassinolide Improves Drought Tolerance in Tobacco Through Physiological and Biochemical Mechanisms.” Biology 11, no. 8: 1192. 10.3390/biology11081192. 36009819 PMC9405153

[pbi70306-bib-0014] Kim, N. Y. , Y. J. Jang , and O. K. Park . 2019. “Corrigendum: AP2/ERF Family Transcription Factors ORA59 and RAP2.3 Interact in the Nucleus and Function Together in Ethylene Response.” Frontiers in Plant Science 10: 42. 10.3389/fpls.2019.00042. 30761175 PMC6363986

[pbi70306-bib-0015] Kong, X. , C. Zhang , H. Zheng , et al. 2020. “Antagonistic Interaction Between Auxin and SA Signaling Pathways Regulates Bacterial Infection Through Lateral Root in Arabidopsis.” Cell Reports 32, no. 8: 108060. 10.1016/j.celrep.2020.108060. 32846118

[pbi70306-bib-0016] Leon‐Reyes, A. , S. H. Spoel , E. S. De Lange , et al. 2009. “Ethylene Modulates the Role of NONEXPRESSOR OF PATHOGENESIS‐RELATED GENES1 in Cross Talk Between Salicylate and Jasmonate Signaling.” Plant Physiology 149, no. 4: 1797–1809. 10.1104/pp.108.133926. 19176718 PMC2663751

[pbi70306-bib-0017] Li, C. , J. Su , N. Zhao , et al. 2023. “CmERF5‐CmRAP2.3 Transcriptional Cascade Positively Regulates Waterlogging Tolerance in *Chrysanthemum morifolium* .” Plant Biotechnology Journal 21, no. 2: 270–282. 10.1111/pbi.13940. 36200911 PMC9884023

[pbi70306-bib-0019] Li, Q. , B. Xian , Q. Yu , et al. 2024. “The CsAP2‐09‐CsWRKY25‐CsRBOH2 Cascade Confers Resistance Against Citrus Bacterial Canker by Regulating ROS Homeostasis.” Plant Journal 118, no. 2: 534–548. 10.1111/tpj.16623. 38230828

[pbi70306-bib-0021] Liu, F. J. , X. H. Qiao , J. H. Hu , et al. 2022. “Resistance Evaluation of Citrus Germplasms Against *Pseuduofabraea Citricarpa* .” Journal of Southwest University Natural Science Edition 44, no. 12: 9–18. 10.13718/j.cnki.xdzk.2022.12.002.

[pbi70306-bib-0020] Liu, F. J. , X. H. Qiao , J. H. Hu , et al. 2023. “Analysis on the Difference of Sensitivity of Two Strains Target Spot Disease Pathogen to Fungicides.” South China Fruits 52, no. 2: 29–35. 10.13938/j.issn.1007-1431.20230127.

[pbi70306-bib-0022] Liu, H. , X. Wang , S. Liu , et al. 2022. “Citrus pan‐Genome to Breeding Database (CPBD): A Comprehensive Genome Database for Citrus Breeding.” Molecular Plant 15, no. 10: 1503–1505. 10.1016/j.molp.2022.08.006. 36004795

[pbi70306-bib-0023] Liu, L. , J. Zhang , J. Xu , et al. 2020. “CRISPR/Cas9 Targeted Mutagenesis of SlLBD40, a Lateral Organ Boundaries Domain Transcription Factor, Enhances Drought Tolerance in Tomato.” Plant Science 301: 110683. 10.1016/j.plantsci.2020.110683. 33218644

[pbi70306-bib-0024] Liu, R. , X. Lv , X. Wang , et al. 2023. “Integrative Analysis of the Multi‐Omics Reveals the Stripe Rust Fungus Resistance Mechanism of the *TaPAL* in Wheat.” Frontiers in Plant Science 14: 1174450. 10.3389/fpls.2023.1174450. 37342140 PMC10277697

[pbi70306-bib-0025] Liu, S. , L. Chen , X. Qiao , J. Ren , C. Zhou , and Y. Yang . 2024. “Functional Evolution of *Pseudofabraea Citricarpa* as an Adaptation to Temperature Change.” Journal of Fungi Basel, Switzerland 10, no. 2: 109. 10.3390/jof10020109. 38392781 PMC10890082

[pbi70306-bib-0026] Liu, Y. , Q. Liu , X. Li , et al. 2023. “MdERF114 Enhances the Resistance of Apple Roots to *Fusarium Solani* by Regulating the Transcription of MdPRX63.” Plant Physiology 192, no. 3: 2015–2029. 10.1093/plphys/kiad057. 36721923 PMC10315273

[pbi70306-bib-0027] Long, Q. , M. Du , J. Long , et al. 2021. “Transcription Factor WRKY22 Regulates Canker Susceptibility in Sweet Orange (*Citrus Sinensis* Osbeck) by Enhancing Cell Enlargement and CsLOB1 Expression.” Horticultural Research 8, no. 1: 50. 10.1038/s41438-021-00486-2. PMC7917094 33642585

[pbi70306-bib-0028] Lorenzo, O. , R. Piqueras , J. J. Sánchez‐Serrano , and R. Solano . 2003. “ETHYLENE RESPONSE FACTOR1 Integrates Signals From Ethylene and Jasmonate Pathways in Plant Defense.” Plant Cell 15, no. 1: 165–178. 10.1105/tpc.007468. 12509529 PMC143489

[pbi70306-bib-0029] Mahavihakanont, A. , N. Charoenlap , P. Namchaiw , et al. 2012. “Novel Roles of SoxR, a Transcriptional Regulator From *Xanthomonas Campestris*, in Sensing Redox‐Cycling Drugs and Regulating a Protective Gene That Have Overall Implications for Bacterial Stress Physiology and Virulence on a Host Plant.” Journal of Bacteriology 194, no. 2: 209–217. 10.1128/JB.05603-11. 22056938 PMC3256661

[pbi70306-bib-0030] Majer, C. , and F. Hochholdinger . 2011. “Defining the Boundaries: Structure and Function of LOB Domain Proteins.” Trends in Plant Science 16, no. 1: 47–52. 10.1016/j.tplants.2010.09.009. 20961800

[pbi70306-bib-0031] Mathelier, A. , O. Fornes , D. J. Arenillas , et al. 2016. “JASPAR: A Major Expansion and Update of the Open‐Access Database of Transcription Factor Binding Profiles.” Nucleic Acids Research 44, no. D1: D110–D115. 10.1093/nar/gkv1176. 26531826 PMC4702842

[pbi70306-bib-0032] McGrath, K. C. , B. Dombrecht , J. M. Manners , et al. 2005. “Repressor‐ and Activator‐Type Ethylene Response Factors Functioning in Jasmonate Signaling and Disease Resistance Identified via a Genome‐Wide Screen of Arabidopsis Transcription Factor Gene Expression.” Plant Physiology 139, no. 2: 949–959. 10.1104/pp.105.068544. 16183832 PMC1256008

[pbi70306-bib-0033] Müller, M. , and S. Munné‐Bosch . 2015. “Ethylene Response Factors: A Key Regulatory Hub in Hormone and Stress Signaling.” Plant Physiology 169, no. 1: 32–41. 10.1104/pp.15.00677. 26103991 PMC4577411

[pbi70306-bib-0034] Nakano, T. , K. Suzuki , T. Fujimura , and H. Shinshi . 2006. “Genome‐Wide Analysis of the ERF Gene Family in Arabidopsis and Rice.” Plant Physiology 140, no. 2: 411–432. 10.1104/pp.105.073783. 16407444 PMC1361313

[pbi70306-bib-0035] Ogawa, T. , L. Pan , M. Kawai‐Yamada , et al. 2005. “Functional Analysis of Arabidopsis Ethylene‐Responsive Element Binding Protein Conferring Resistance to Bax and Abiotic Stress‐Induced Plant Cell Death.” Plant Physiology 138, no. 3: 1436–1445. 10.1104/pp.105.063586. 15980186 PMC1176415

[pbi70306-bib-0036] Papdi, C. , I. Pérez‐Salamó , M. P. Joseph , et al. 2015. “The Low Oxygen, Oxidative and Osmotic Stress Responses Synergistically Act Through the Ethylene Response Factor VII Genes RAP2.12, RAP2.2 and RAP2.3.” Plant Journal 82, no. 5: 772–784. 10.1111/tpj.12848. 25847219

[pbi70306-bib-0037] Pré, M. , M. Atallah , A. Champion , M. De Vos , C. M. Pieterse , and J. Memelink . 2008. “The AP2/ERF Domain Transcription Factor ORA59 Integrates Jasmonic Acid and Ethylene Signals in Plant Defense.” Plant Physiology 147, no. 3: 1347–1357. 10.1104/pp.108.117523. 18467450 PMC2442530

[pbi70306-bib-0038] Qiu, W. , J. Soares , Z. Pang , et al. 2020. “Potential Mechanisms of *AtNPR1* Mediated Resistance Against Huanglongbing (HLB) in Citrus.” International Journal of Molecular Sciences 21, no. 6: 2009. 10.3390/ijms21062009. 32187998 PMC7139736

[pbi70306-bib-0039] Sato, M. , K. Tsuda , L. Wang , et al. 2010. “Network Modeling Reveals Prevalent Negative Regulatory Relationships Between Signaling Sectors in Arabidopsis Immune Signaling.” PLoS Pathogens 6, no. 7: e1001011. 10.1371/journal.ppat.1001011. 20661428 PMC2908620

[pbi70306-bib-0040] Shi, W. , Y. Song , T. Liu , et al. 2021. “StRAP2.3, an ERF‐VII Transcription Factor, Directly Activates StInvInh2 to Enhance Cold‐Induced Sweetening Resistance in Potato.” Horticultural Research 8, no. 1: 82. 10.1038/s41438-021-00522-1. PMC8012585 33790269

[pbi70306-bib-0041] Shuai, B. , C. G. Reynaga‐Peña , and P. S. Springer . 2002. “The Lateral Organ Boundaries Gene Defines a Novel, Plant‐Specific Gene Family.” Plant Physiology 129, no. 2: 747–761. 10.1104/pp.010926. 12068116 PMC161698

[pbi70306-bib-0042] Singh, K. , R. C. Foley , and L. Oñate‐Sánchez . 2002. “Transcription Factors in Plant Defense and Stress Responses.” Current Opinion in Plant Biology 5, no. 5: 430–436. 10.1016/s1369-5266(02)00289-3. 12183182

[pbi70306-bib-0043] Thatcher, L. F. , J. J. Powell , E. A. Aitken , K. Kazan , and J. M. Manners . 2012. “The Lateral Organ Boundaries Domain Transcription Factor LBD20 Functions in Fusarium Wilt Susceptibility and Jasmonate Signaling in Arabidopsis.” Plant Physiology 160, no. 1: 407–418. 10.1104/pp.112.199067. 22786889 PMC3440215

[pbi70306-bib-0044] Timmer, L. W. , H. M. Darhower , S. E. Zitko , T. L. Peever , A. M. Ibáñez , and P. M. Bushong . 2000. “Environmental Factors Affecting the Severity of Alternaria Brown Spot of Citrus and Their Potential Use in Timing Fungicide Applications.” Plant Disease 84, no. 6: 638–643. 10.1094/PDIS.2000.84.6.638. 30841103

[pbi70306-bib-0045] Wang, J. W. , J. X. Wang , Y. Zhu , et al. 2023. “Building an Improved Transcription Factor‐Centered Yeast One Hybrid System to Identify DNA Motifs Bound by Protein Comprehensively.” BMC Plant Biology 23, no. 1: 236. 10.1186/s12870-023-04241-8. 37142946 PMC10158250

[pbi70306-bib-0046] Wang, N. , F. Chi , Z. Ji , Z. Zhou , and J. Zhang . 2021. “First Report of Passion Fruit Anthracnose Caused by *Colletotrichum Constrictum* .” Plant Disease 22: 4158. 10.1094/PDIS-04-21-0754-PDN.

[pbi70306-bib-0047] Wang, Z. , R. Zhang , Y. Cheng , et al. 2021. “Genome‐Wide Identification, Evolution, and Expression Analysis of LBD Transcription Factor Family in Bread Wheat (*Triticum Aestivum* L.).” Frontiers in Plant Science 12: 721253. 10.3389/fpls.2021.721253. 34539714 PMC8446603

[pbi70306-bib-0048] Xiao, X. , Y. Zeng , W. Wang , L. Cheng , and H. Li . 2020. “First Report and New Hosts of *Pseudofabraea Citricarpa* Causing Citrus Target Spot in China.” Plant Health Progress 22, no. 1: 1–5. 10.1094/PHP-07-20-0056-RS.

[pbi70306-bib-0049] Yang, L. , Q. Sun , B. Geng , et al. 2023. “Jasmonate Biosynthesis Enzyme allene Oxide Cyclase 2 Mediates Cold Tolerance and Pathogen Resistance.” Plant Physiology 193, no. 2: 1621–1634. 10.1093/plphys/kiad362. 37392433

[pbi70306-bib-0050] Yang, Y. N. , Y. Kim , H. Kim , et al. 2021. “The Transcription Factor ORA59 Exhibits Dual DNA Binding Specificity That Differentially Regulates Ethylene‐ and Jasmonic Acid‐Induced Genes in Plant Immunity.” Plant Physiology 187, no. 4: 2763–2784. 10.1093/plphys/kiab437. 34890461 PMC8644270

[pbi70306-bib-0051] Zafar, M. M. , A. Rehman , A. Razzaq , et al. 2022. “Genome‐Wide Characterization and Expression Analysis of erf Gene Family in Cotton.” BMC Plant Biology 22, no. 1: 134. 10.1186/s12870-022-03521-z. 35317739 PMC8939120

[pbi70306-bib-0053] Zhan, S. , W. Wu , J. Hu , et al. 2021. “Pathogen Identification and Screening of Control Agents for Suspected Citrus Target Spot in Wanzhou of Chongqing.” South China Fruits 1, no. 1: 1–7.

[pbi70306-bib-0052] Zhan, S. , W. Wu , J. Hu , et al. 2024. “The Pathogenicity and Regulatory Function of Temperature‐Sensitive Proteins *PscTSP* in *Pseudofabraea Citricarpa* Under High Temperature Stress.” International Journal of Biological Macromolecules 270, no. Pt1: 132017. 10.1016/j.ijbiomac.2024.132017. 38697438

[pbi70306-bib-0054] Zhang, D. , K. Zhu , X. Shen , et al. 2023. “Two Interacting Ethylene Response Factors Negatively Regulate Peach Resistance to *Lasiodiplodia Theobromae* .” Plant Physiology 192, no. 4: 3134–3151. 10.1093/plphys/kiad279. 37165714 PMC10400035

[pbi70306-bib-0055] Zhang, M. , S. Huang , Y. Gao , et al. 2020. “Fine Mapping of a Leaf Flattening Gene Bralcm Through BSR‐Seq in Chinese Cabbage (*Brassica Rapa* L. Ssp. Pekinensis).” Scientific Reports 10, no. 1: 13924. 10.1038/s41598-020-70975-2. 32811880 PMC7435182

[pbi70306-bib-0056] Zhu, L. , X. Wang , F. Huang , et al. 2012. “A Destructive New Disease of Citrus in China Caused by *Cryptosporiopsis Citricarpa* Sp.” Plant Disease 96, no. 6: 804–812. 10.1094/PDIS-09-11-0775. 30727352

[pbi70306-bib-0057] Zhu, X. , L. Qi , X. Liu , et al. 2014. “The Wheat Ethylene Response Factor Transcription Factor Pathogen‐Induced ERF1 Mediates Host Responses to Both the Necrotrophic Pathogen *Rhizoctonia Cerealis* and Freezing Stresses.” Plant Physiology 164, no. 3: 1499–1514. 10.1104/pp.113.229575. 24424323 PMC3938636

[pbi70306-bib-0058] Zou, X. , M. Du , Y. Liu , et al. 2021. “ *CsLOB1* Regulates Susceptibility to Citrus Canker Through Promoting Cell Proliferation in Citrus.” Plant Journal 106, no. 4: 1039–1057. 10.1111/tpj.15217. 33754403

