# Supplementary figures and images for: The CsLOB1‐CsERF027 Regulatory Module Positively Enhances Citrus Target Spot Disease Resistance by Regulating CsRAP2.3‐CsERF1 Cascade
Source: Plant Biotechnol J. 2025 Aug 28;23(12):5745–61. doi: 10.1111/pbi.70306 (PMC12665070; doi:10.1111/pbi.70306)

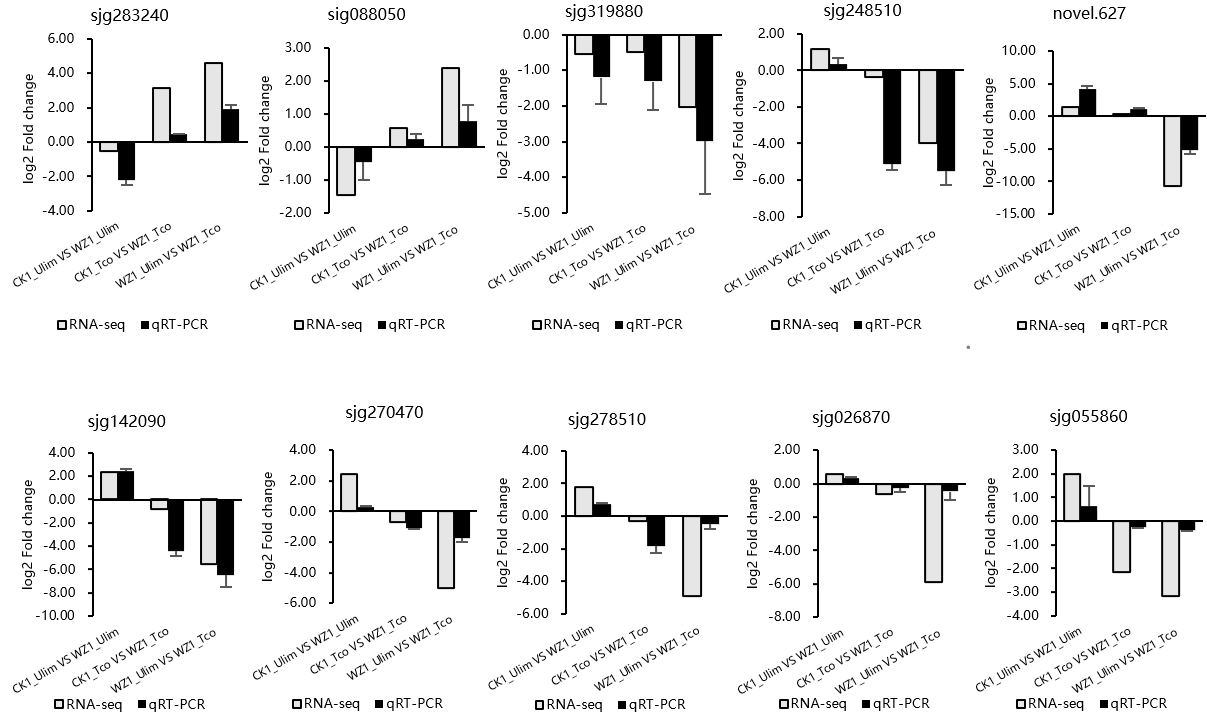


**C**


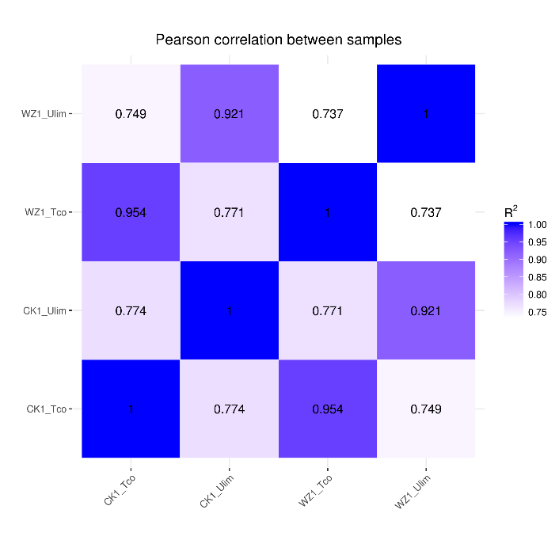


**A**


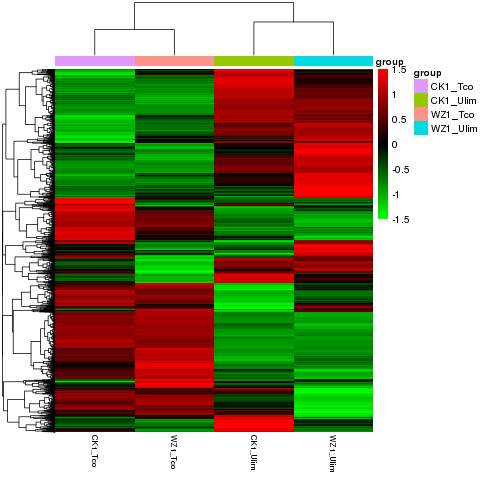


**B**


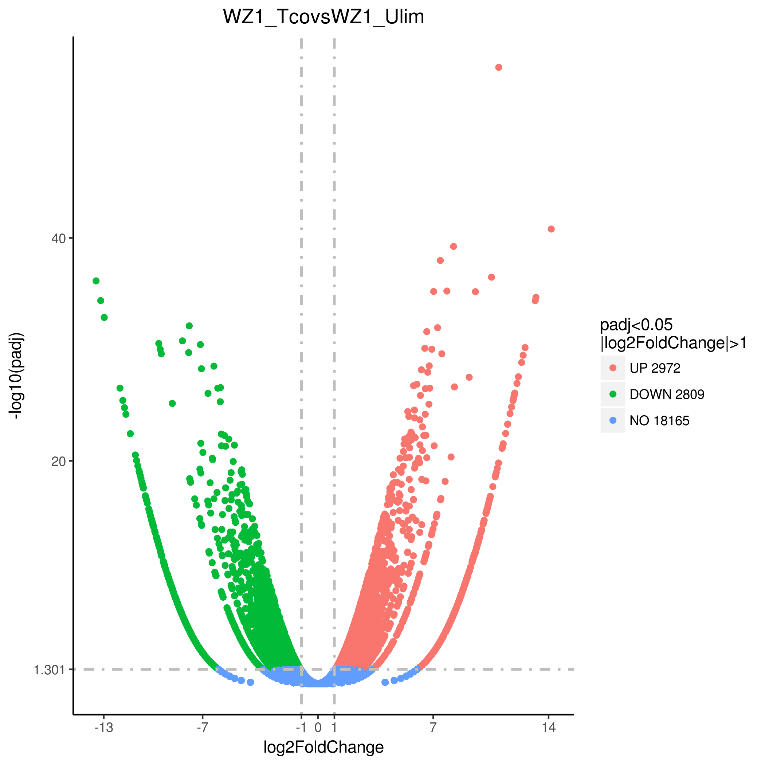


**D**


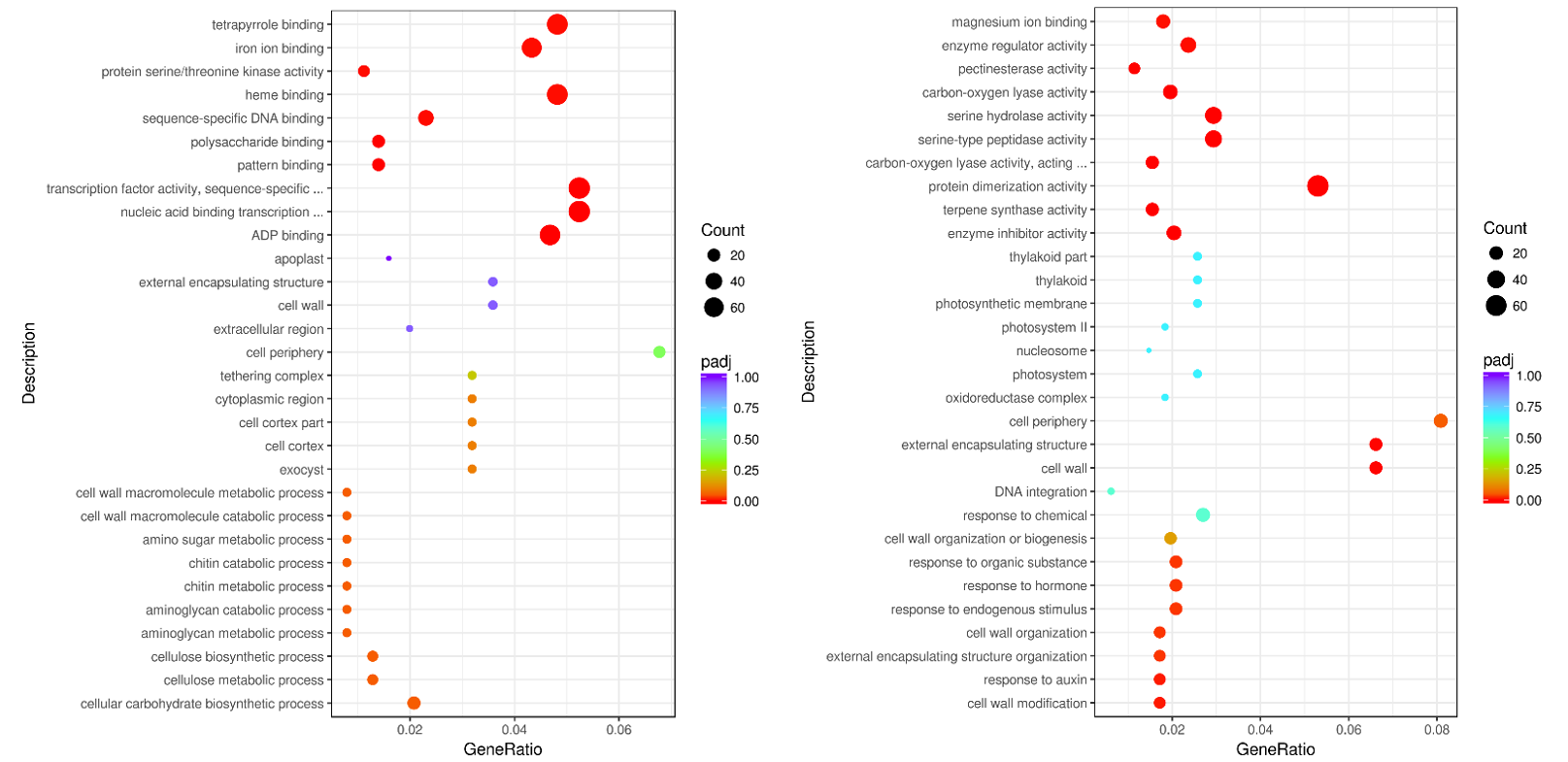


**E**

**F**


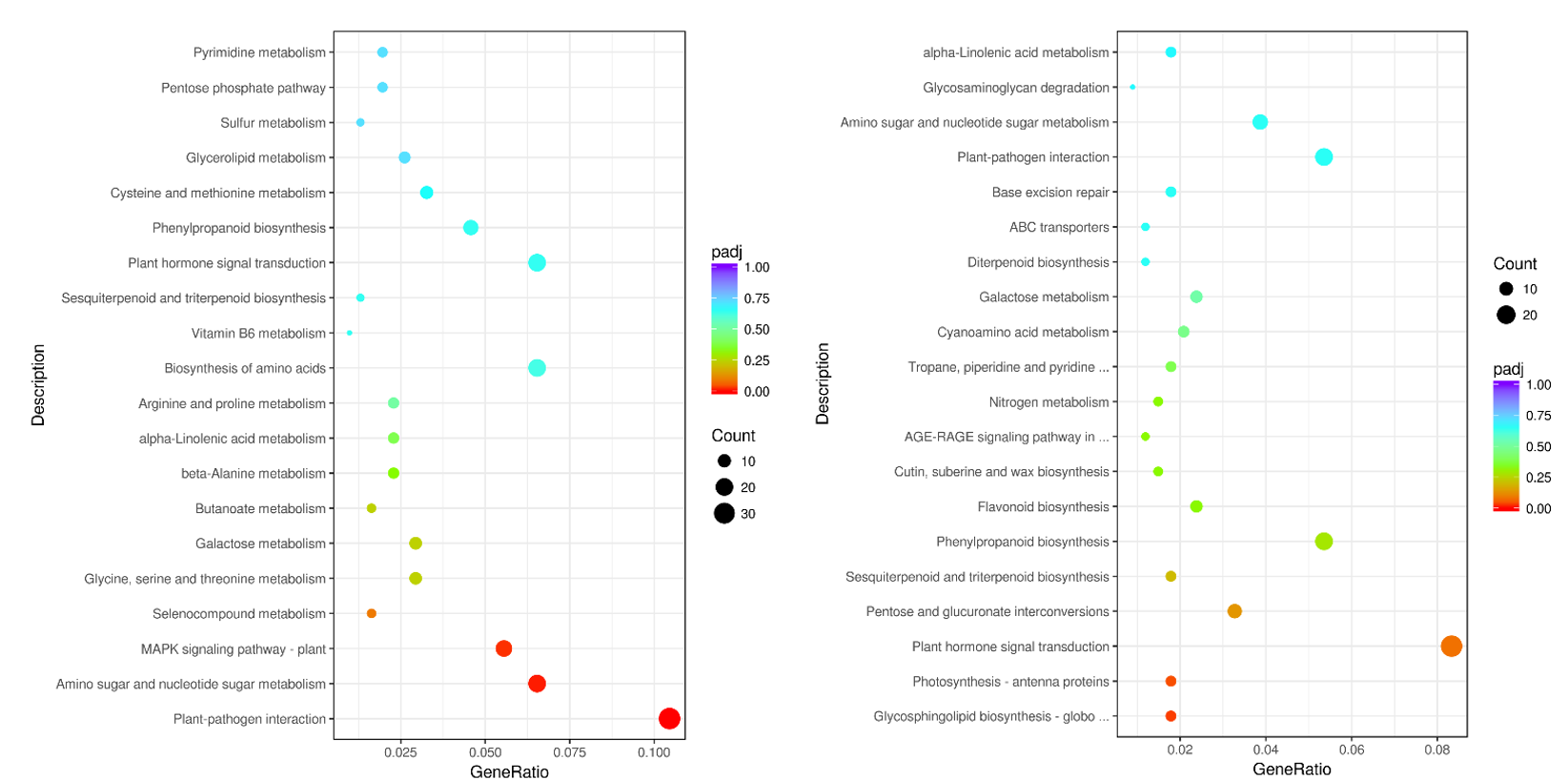


**G**

**H**

Supplement: Supplementary file 1 — Figure S1: Correlation analysis and enrichment pathway analysis of RNA‐Seq data. (A) Pearson correlation coefficient. (B) Cluster results for four samples. (C) Reliability results of RT‐qPCR‐verified RNA‐Seq data. (D) Differentially expressed gene analysis of WZ1_Tco VS WZ1_Ulim. (E) GO enrichment analysis of upregulated genes in WZ1_Tco VS WZ1_Ulim. (F) GO enrichment analysis of downregulated genes in WZ1_Tco VS WZ1_Ulim. (G) KEGG enrichment analysis of upregulated gene expression in WZ1_Tco VS WZ1_Ulim. (F) KEGG enrichment analysis of downregulated gene expression in WZ1_Tco VS WZ1_Ulim. [file PBI-23-5745-s001.docx]
